# Proteomic and metabolomic insights into the impact of topping treatment on cigar tobacco

**DOI:** 10.3389/fpls.2024.1425154

**Published:** 2025-02-20

**Authors:** Dong Guo, Huajun Gao, Tongjing Yan, Changjian Xia, Beisen Lin, Xiaohua Xiang, Bin Cai, Zhaoliang Geng

**Affiliations:** Haikou Cigar Research Institute, Hainan Province Company, China National Tobacco Corporation, Haikou, China

**Keywords:** tobacco, top removal, flavonoid biosynthesis, proteomics, metabolomics

## Abstract

Top removal is a widely utilized method in production process of tobacco, but little is known regarding the way it impacts protein and metabolic regulation. In this study, we investigated the underlying processes of alterations in cigar tobacco leaves with and without top removal, using a combined proteomic and metabolomic approach. The results revealed that: (1) Topping significantly affected superoxide anion (O_2_^-^) levels, superoxide dismutase (SOD) activity, and malondialdehyde (MDA) content, (2) In the cigar tobacco proteome, 385 differentially expressed proteins (DEPs) were identified, with 228 proteins upregulated and 156 downregulated. Key pathways enriched included flavonoid biosynthesis, porphyrin and chlorophyll metabolism, cysteine and methionine metabolism, and amino acid biosynthesis and metabolism. A network of 161 nodes interconnected by 102 significantly altered proteins was established, (3) In the cigar tobacco metabolome, 247 significantly different metabolites (DEMs) were identified, with 120 upregulated and 128 downregulated metabolites, mainly comprising lipids and lipid-like molecules, phenylpropanoids and polyketides, organic acids and derivatives, and organic heterocyclic compounds, (4) KEGG pathway enrichment revealed upregulation of proteins such as chalcone synthase (CHS), chalcone isomerase (CHI), naringenin 3-dioxygenase (F3H), and flavonoid 3’-monooxygenase (F3’H), along with metabolites like pinocembrin, kaempferol, trifolin, rutin, and quercetin, enhancing the pathways of ‘flavonoid’ and ‘flavone and flavonol’ biosynthesis. This study sheds light on the metabolic and proteomic responses of cigar tobacco after topping.

## 1 Introduction

Tobacco is an important economic crop cultivated over vast expanses (Chen et al., 2021). In China, it is categorized into four types based on drying techniques and agronomic traits: flue-cured, sun-cured, air-cured, and burley tobacco (Liu et al., 2021). Cigar tobacco, an air-cured variety, is one of the most commonly grown and traditional types of tobacco. A cigar consists of three main components: the wrapper, binder, and filler, all made from tobacco leaves (Zhang et al., 2023a). As a result, the quality and yield of tobacco leaves are exceedingly significant. In tobacco cultivation, topping—the removal of the plant’s upper portion—is a key agronomic practice that plays a crucial role in enhancing both the quality and quantity of the leaves (Baldwin, 2001; Qin et al., 2020).

Topping in plants, particularly in tobacco, involves the removal of the upper flowering parts and young leaves and is an essential component of tobacco farming (Dai et al., 2022). As tobacco plants mature, they progress from the vegetative to the reproductive phase, with the apical meristem of the main stem converting into floral meristems. When the plant begins flowering, it diverts a significant amount of nutrients to the top, creating a reproductive growth zone. This process involves the movement of water and nutrients from the roots, along with the redistribution of resources from the lower and middle leaves to the upper parts (Shi et al., 2006). This nutrient redistribution becomes increasingly evident as tobacco flowers and seeds develop (Geuns et al., 2001). Nutrients generated in flue-cured tobacco leaves are primarily directed toward flowering, which limits leaf growth. This results in smaller, lighter upper leaves, diminishing the overall quality and yield (Chen et al., 2019). When carried out at the appropriate moment, it redirects the plant’s energy toward the remaining leaves, improving their yield and quality (Yan et al., 2019; Lei et al., 2022). This technique shifts the plant’s primary growth and nutrient distribution centers, altering the original source-sink relationship (Ogilvie and Kozumplik, 1980). Consequently, it influences several biological functions, including secondary metabolism and hormone regulation (Guo et al., 2023). Topping substantially elevates the levels of secondary metabolites like nicotine and aromatic amines (Shi et al., 2006; Banožić et al., 2020), and enhances the expression of genes linked to hormone metabolism and plant defense (Guo et al., 2011). Essentially a form of mechanical damage, topping mimics the effects of insect attacks or physical injuries, leading to an accumulation of reactive oxygen species (ROS), which cause oxidative stress (Gaquerel et al., 2009; Luo et al., 2016; Yan et al., 2019). This activates the plant’s defense mechanisms, prompting the production of secondary metabolites and volatile compounds such as alkaloids and carotenoids. Jasmonic acid (JA) plays a central role in this defense response, regulating carbohydrate distribution and secondary metabolite formation (Katoh et al., 2007; Deboer et al., 2009; Ma et al., 2019), thereby increasing antimicrobial compounds. Therefore, topping is a crucial field management practice during tobacco plant maturation. However, research on cigar tobacco’s proteomic and metabolomic responses to topping remains incomplete.

With the ongoing advancement and adoption of omics technologies, proteomics and metabolomics offer deeper insights into tobacco proteins and their metabolites (Wang et al., 2021). Proteomics, through protein quantification, links gene transcription to metabolic processes (Jin et al., 2015; Cao et al., 2024), and combining proteomics with transcriptomics improves the understanding of regulatory networks. Metabolomics, a vital element of systems biology, identifies a broad spectrum of endogenous metabolites (Wang et al., 2019; Yang et al., 2024). But little is known regarding the way it impacts protein and metabolic regulation, this study employs proteomic and metabolomic analyses to reveal the antioxidant response mechanisms and regulatory processes governing physiological changes in cigar tobacco after topping.

## 2 Materials and methods

### 2.1 Plant materials and treatment

The ‘Haiyan103’ variety of cigar tobacco, provided by the Haikou Cigar Research Institute in Haikou, China, was the subject of our investigation. Our goal was to evaluate how topping—removal of the top floral portion and upper young leaves—affects the growth of tobacco plants. We chose plants from Danzhou, one of the main tobacco-growing regions in the Hainan Province. The study included two groups: plants without topping (DDW1) and plants with topping (DDW2). Topping was performed 55 days after transplanting, and samples were collected 5 days later. The untopped plants served as the control group, with three replicates in each plot.

Plants were arranged in rows 1.2 meters apart, with 0.6 meters between individual plants. Each treatment received 180 kg of N, 270 kg of P_2_O_5_, and 360 kg of K_2_O per hectare, and was managed according to standard cigar tobacco cultivation practices (Lei et al., 2014).

During sampling, nine plants were chosen at random from each group as a biological duplicate. On cigar tobacco plants, the eighth leaf (counted from base to tip, undamaged leaves with an area of approximately 48 × 26 cm) was chosen as the sample, and the leaf’s central vein was removed. The leaves were then pooled, wrapped in foil, and quickly frozen in liquid nitrogen. For each biological replicate, we ensured sample uniformity by freezing and grinding the samples in liquid nitrogen.

### 2.2 Antioxidant index evaluation

To assess superoxide dismutase (SOD) activity, we prepared the enzyme solution according to the procedure outlined by Durak et al. (1993). SOD activity was quantified spectrophotometrically at 560 nm, based on its ability to inhibit the 50% reduction of nitro blue tetrazolium (NBT). The superoxide anion (O_2_^-^) levels were measured using the hydroxylamine oxidation method described by Zhuang et al. (2019). Malondialdehyde (MDA) levels were determined using the thiobarbituric acid (TBA) reactive substances method, as detailed by Draper et al. (1993).

### 2.3 Proteomic analysis

#### 2.3.1 Protein isolation and digestion process

Protein isolation was carried out by grinding 0.5 g of tobacco leaf tissue into a powder in liquid nitrogen. The powder was then swiftly transferred to a pre-cooled centrifuge tube containing 800 µL of SDT lysis buffer (with 100 mM NaCl) and 1/100 volume of dithiothreitol (DTT). The mixture was shaken thoroughly and sonicated in an ice bath for 5 minutes to lyse the sample completely. It was then heated at 95°C for 8-15 minutes, cooled in an ice bath for 2 minutes, and centrifuged at 4°C at 12,000 g for 15 minutes. To the supernatant, 80 µL of iodoacetamide (IAM) solution was added and kept in the dark (in a closed drawer) for 1 hour. Next, 2 mL of pre-cooled acetone was added and the mixture was precipitated at -20°C for 2 hours, followed by centrifugation at 4°C at 12,000 g for 15 minutes, and the resultant precipitate was collected. The precipitate was then suspended and washed in 1 mL of pre-cooled acetone, collected, and air-dried. The resulting precipitate, which represents the total protein, was then dissolved in 1 mL of Dissolved Buffer (DB buffer).

Protein digestion was performed as outlined by Zhang et al. (2016). A total of 200 µL of DB protein dissolution buffer was added. Then, 2 µg of trypsin and 100 mM Triethylammonium bicarbonate (TEA) buffer were added, mixed thoroughly, and incubated at 37°C for 4 hours. Afterward, another 2 µg of trypsin and 200 mM CaCl_2_ were added, followed by overnight incubation at 37°C for further enzymatic digestion. Formic acid was used to lower the pH below 3, and then the mixture was centrifuged at 12,000 g for 5 minutes at room temperature. The supernatant was slowly passed through a C18 desalting column, washed thrice with a solution of 0.1% formic acid and 3% acetonitrile, and eluted with 200 µL of elution solution (0.1% formic acid, 70% acetonitrile). The filtrate was then collected and lyophilized.

#### 2.3.2 Proteomic analysis via UHPLC-MS/MS

For proteomic analysis, we employed an Easy-nLC™ 1200 UHPLC (Thermo Fisher, Germany) coupled with a Q Exploris™ HF-X mass spectrometer (Thermo Fisher, Germany). A total of 4 μg of each sample, combined with iRT reagent, was loaded onto a C18 Nano-Trap column. The gradient profile ranged from 5% to 95% acetonitrile in formic acid over 92 minutes, at a flow rate of 600 nL/min. Peptide analysis was performed using a Q Exactive™ HF-X mass spectrometer with specific settings for full scan range, resolution, AGC target, ion injection time, and fragmentation. The top 40 precursors were selected for MS/MS analysis, with DIA mode applied for broader peptide coverage.

#### 2.3.3 Identification and quantification of proteins

We individually analyzed the spectra from each fraction against the *Nicotiana tabacum* protein database using Proteome Discoverer 2.2. The search parameters were carefully optimized for precision and identified proteins were required to satisfy strict criteria, including FDR and amino acid coverage.

#### 2.3.4 Data analysis and statistical approaches

For functional annotation, we used Gene Ontology and InterPro analyses through InterProScan, comparing against an extensive protein database. Additionally, we utilized the COG and KEGG databases to further analyze protein families and pathways. Differential protein expression was examined with various tools, including Volcano plot and heat map analysis. Protein-protein interactions were predicted using the STRING-db server.

### 2.4 Metabolomics analysis

#### 2.4.1 Metabolite extraction and untargeted metabolomic analysis

0.1 g of leaf tissue was ground in liquid nitrogen to form a fine powder, which was then reconstituted in pre-chilled 80% methanol. After vigorously shaking the mixture, it was placed on ice for 5 minutes and then centrifuged at 15,000 g for 20 minutes at 4°C. The clear supernatant was diluted to a 53% methanol concentration using UHPLC-MS/MS grade water. The diluted mixture was transferred to new Eppendorf tubes and centrifuged again under the same conditions. The resulting supernatant was then prepared for UHPLC-MS/MS analysis.

The samples were analyzed using a Vanquish UHPLC system (Thermo Fisher, Germany), paired with an Orbitrap Q Exactive™ HF mass spectrometer (Thermo Fisher, Germany). The samples were loaded onto a Hypersil Gold column (100 × 2.1 mm, 1.9 μm)(Thermo Fisher, USA) using a 12-minute linear gradient at a flow rate of 0.2 mL/min. For positive polarity mode, eluent A was 0.1% formic acid in water and eluent B was methanol. For negative polarity mode, eluent A was 5 mM ammonium acetate (pH 9) and eluent B was methanol. The solvent gradient was: 2% B, 1.5 minutes; 2-85% B, 3 minutes; 85-100% B, 10 minutes; 100-2% B, 10.1 minutes; 2% B, 12 minutes. The mass spectrometer parameters, including spray voltage, capillary temperature, gas flow rates, and S-lens RF level, were precisely calibrated. The MS scan range was 90 to 900 m/z, with fragmentation data acquisition in both polarity modes.

#### 2.4.2 Processing and analysis of metabolomics data

To process the metabolomics data, we employed Compound Discoverer 3.3. The metabolite identification process involved comparing the acquired data against various databases, including mzCloud, mzVault, KEGG, and others, while adhering to a strict mass tolerance. For metabolite annotation, we relied on established databases such as KEGG, HMDB, and LIPID Maps, which provide a wealth of information for precise identification and classification.

#### 2.4.3 Synthesis of proteomic and metabolomic data

The incorporation of the identified differentially expressed proteins (DEPs) and metabolites (DEMs) into the KEGG pathway maps enabled the visualization of changes in key metabolic pathways.

### 2.5 Statistical analysis

Data preparation was carried out with Microsoft Excel 2023, and all statistical analyses were conducted in SPSS V16.0 for Windows (SPSS, Chicago, Illinois, USA). A difference was deemed statistically significant if P < 0.05.

## 3 Results

### 3.1 Effect of topping on antioxidant enzymes

**Figure 1:**
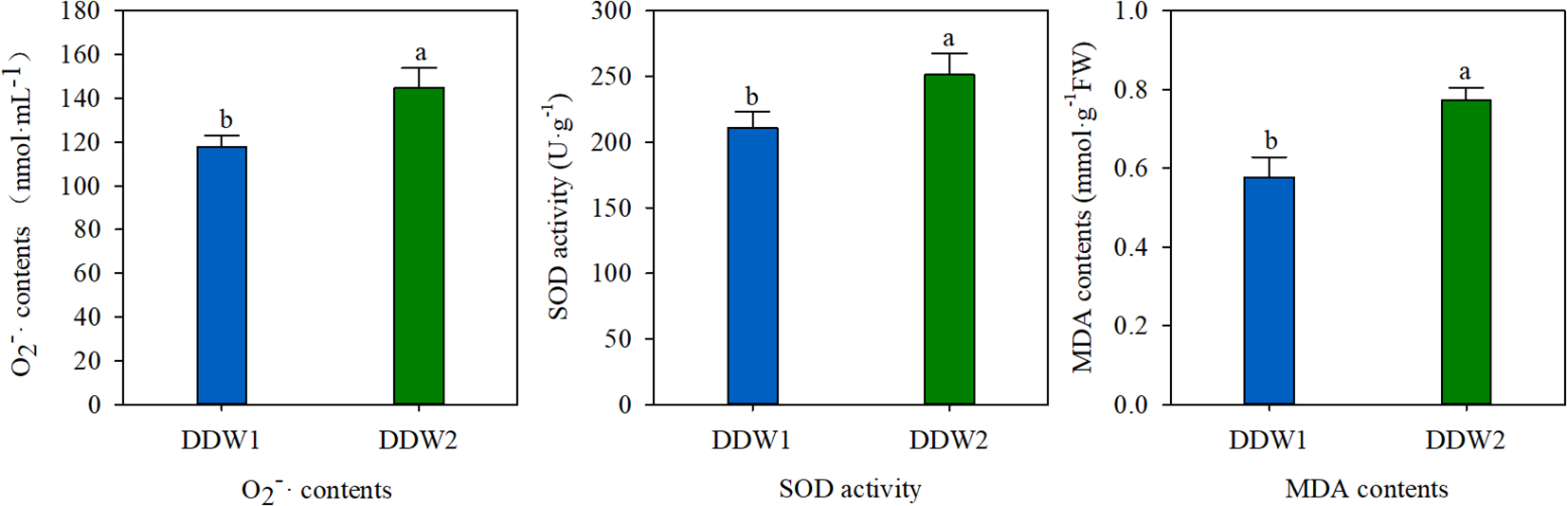
That topping has an effect on O_2_^-^ content, SOD activity and MDA content. Different letters above the error bars indicate significant difference at the 0.05 probability level.

The antioxidant enzyme indices of tobacco leaves that underwent topping were compared to those that did not. As illustrated in **Figure 1**, topping had a significant effect on O_2_^-^ content, SOD activity, and MDA content. Specifically, O_2_^-^ levels in the DDW2 group were 22.95% higher than in the DDW1 group. In a similar vein, SOD activity in the DDW2 group was 19.30% higher than in the DDW1 group. Furthermore, MDA content in the DDW2 group was 34.10% higher, demonstrating a statistically significant difference.

### 3.2 Identification of proteins

**Figure 2:**
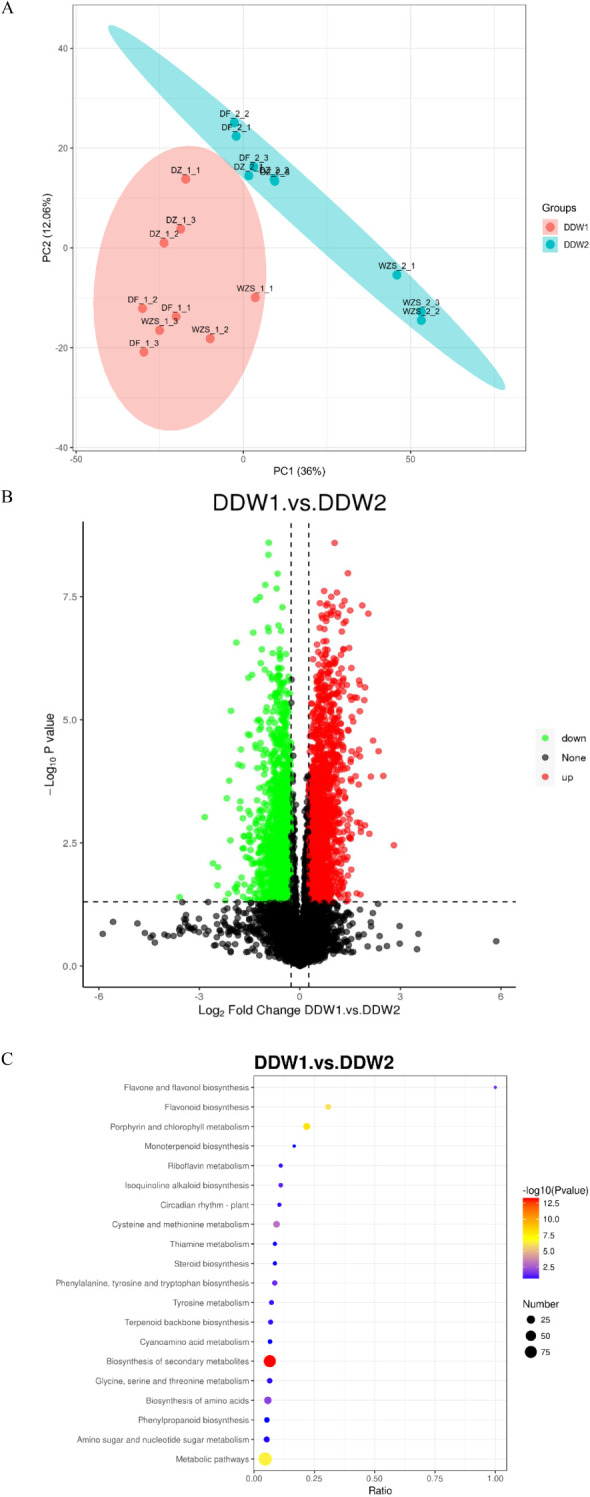
Identification of Proteins. **(A)** PCA result of proteins obtained; **(B)** Volcano map of differential proteins; **(C)** KEGG pathway enrichment result of differential proteins.

An unsupervised, multi-factor principal component analysis (PCA) was performed to evaluate the protein profile data of cigar tobacco leaves subjected to topping and non-topping treatments. As depicted in **Figure 1**, the two groups of cigar tobacco leaves were significantly separated along the first principal component, with minimal dispersion among samples within each treatment group. All samples fell within the 95% confidence interval, indicating significant differences in protein profiles between the DDW1 and DDW2 groups. Analysis using the Proteome Discoverer (PD) software confirmed high sample repeatability in this study, as illustrated in **Figure 2A**. The proteomic analysis of cigar tobacco leaves in the DDW1 and DDW2 groups identified a total of 12,677 proteins. All proteins in this investigation were subjected to screening and analysis utilizing a threshold of VIP > 1, FC > 1.5 or FC < 0.67, and P < 0.05. The results demonstrated that, compared to the DDW2 group, the DDW1 group had 385 DEPs, with 229 up-regulated and 156 down-regulated (**Figure 2B**; **Supplementary Table 2**). KEGG pathway analysis, conducted using KOBAS 3.0 online software, revealed the top 20 enriched pathways among these DEPs (**Figure 2C**). The significantly enriched pathways included flavone and flavonol biosynthesis, flavonoid biosynthesis, porphyrin and chlorophyll metabolism, cysteine and methionine metabolism, amino acid biosynthesis, and general metabolic pathways.

### 3.3 Protein-protein interaction (PPI) analysis of Differentially Expressed Proteins (DEPs)

**Figure 3:**
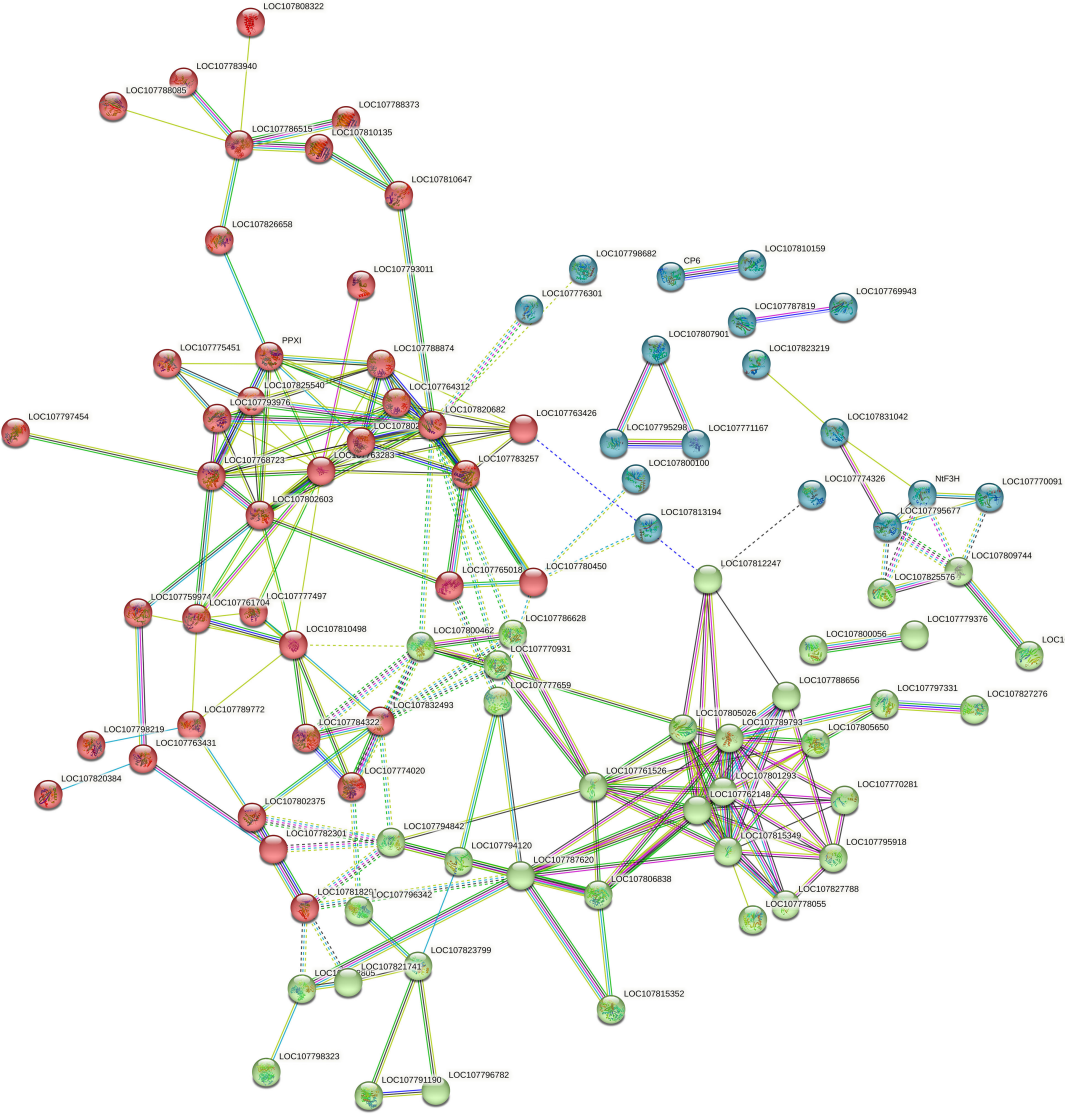
The PPI analysis of differentially expressed proteins.

Protein-protein interaction networks were generated using the publicly accessible STRING database, revealing a network of 161 nodes interconnected by 102 interactions among significantly altered proteins. Cluster analysis within STRING showed that these networks were mainly composed of proteins involved in key metabolic pathways. These included porphyrin and chlorophyll metabolism, seleno-compound metabolism, amino sugar and nucleotide sugar metabolism, biosynthesis of phenylalanine, tyrosine, and tryptophan, flavonoid biosynthesis, glyoxylate and dicarboxylate metabolism, amino acid biosynthesis, flavone and flavonol biosynthesis, as well as steroid and secondary metabolites biosynthesis. The results are illustrated in **Figure 3** and detailed in **Supplementary Table 1**.

### 3.4 Identification and analysis of metabolites

**Figure 4:**
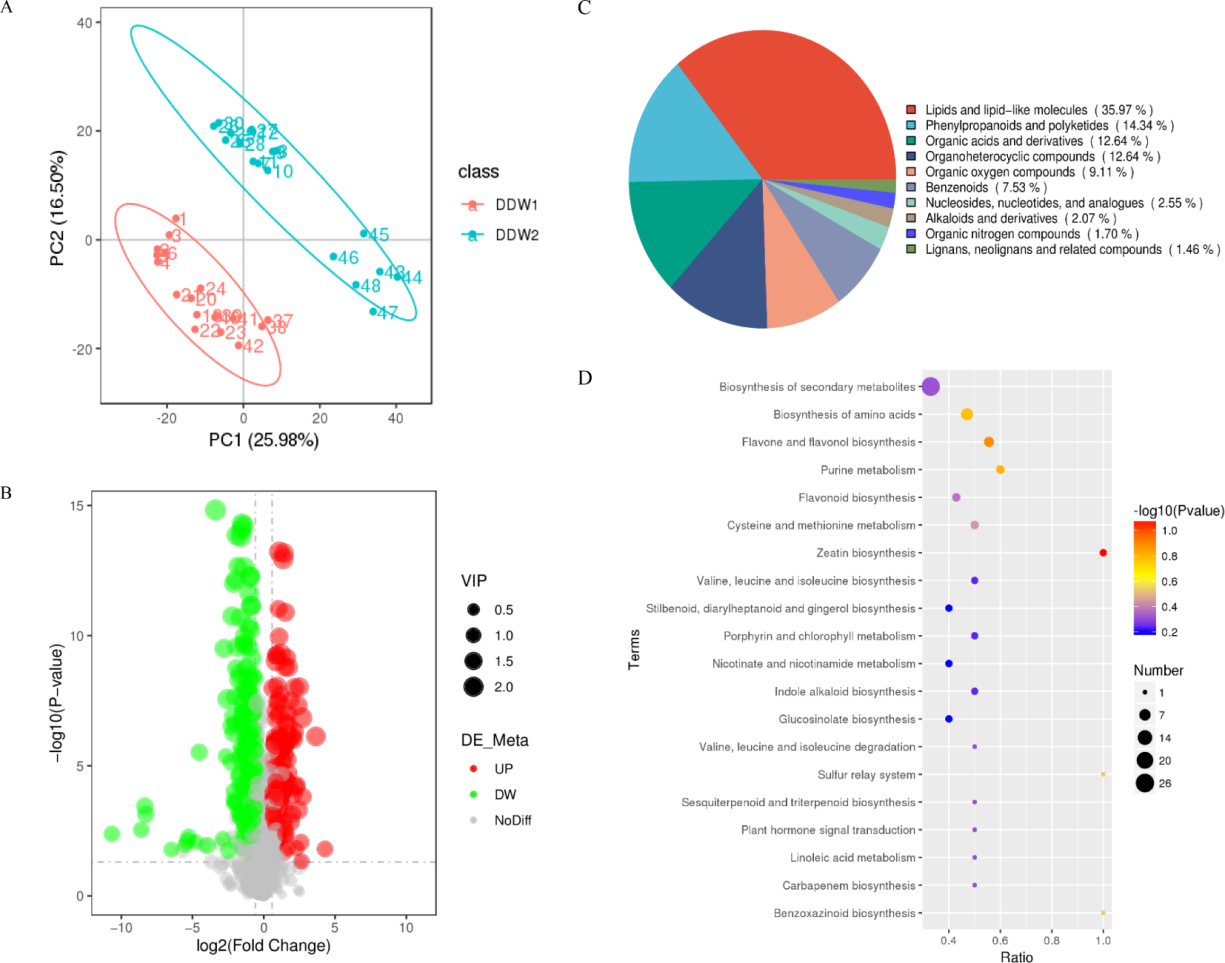
Identification and Analysis of Metabolites. **(A)** PCA result of metabolites obtained; **(B)** Volcano map of differential metabolites; **(C)** the pie chart shows the category results of the differential metabolites; **(D)** KEGG pathway enrichment result of differential proteins.

The PCA results are displayed in **Figure 4A**. Samples from the DDW1 and DDW2 groups were significantly separated along the first and second principal components, with little variation within each treatment group. All samples fell within the 95% confidence interval, highlighting significant differences in the metabolite profiles of cigar tobacco leaves between the two treatment groups. Through the metabolic analysis of cigar tobacco leaves in the DDW1 and DDW2 groups, a total of 1163 metabolites were quantified. All metabolites in this investigation were screened and examined using a threshold of VIP > 1, FC > 1.2 or FC < 0.83, and P < 0.05. The results demonstrated that, compared to the DDW1 group, the cigar tobacco leaves in the DDW2 group had 247 DEMs, with 120 up-regulated and 128 down-regulated (**Figure 4B**; **Supplementary Table 3**). The bulk of these differential metabolites were composed of lipids and lipid-like molecules, phenylpropanoids and polyketides, organic acids and derivatives, and organoheterocyclic compounds, as depicted in **Figure 4C**. Pathway enrichment analysis highlighted eight key enriched pathways among these metabolites, including zeatin biosynthesis, sulfur relay system, benzoxazinoid biosynthesis, purine metabolism, flavone and flavonol biosynthesis, amino acid biosynthesis, cysteine and methionine metabolism, and flavonoid biosynthesis, shown in **Figure 4D**. Additionally, correlation analysis revealed that 2-hydroxyisocaproic acid had a significant positive correlation with gamma-caprolactone and octanedioic acid. Sattabacin showed a significant positive correlation with (±)-abscisic acid and methyl jasmonate, and a positive correlation was also observed between octanedioic acid and gamma-caprolactone with methyl jasmonate and (±)-abscisic acid, respectively, as detailed in **Supplementary Figure 1**.

### 3.5 Merging proteomic and metabolomic data studies

**Figure 5:**
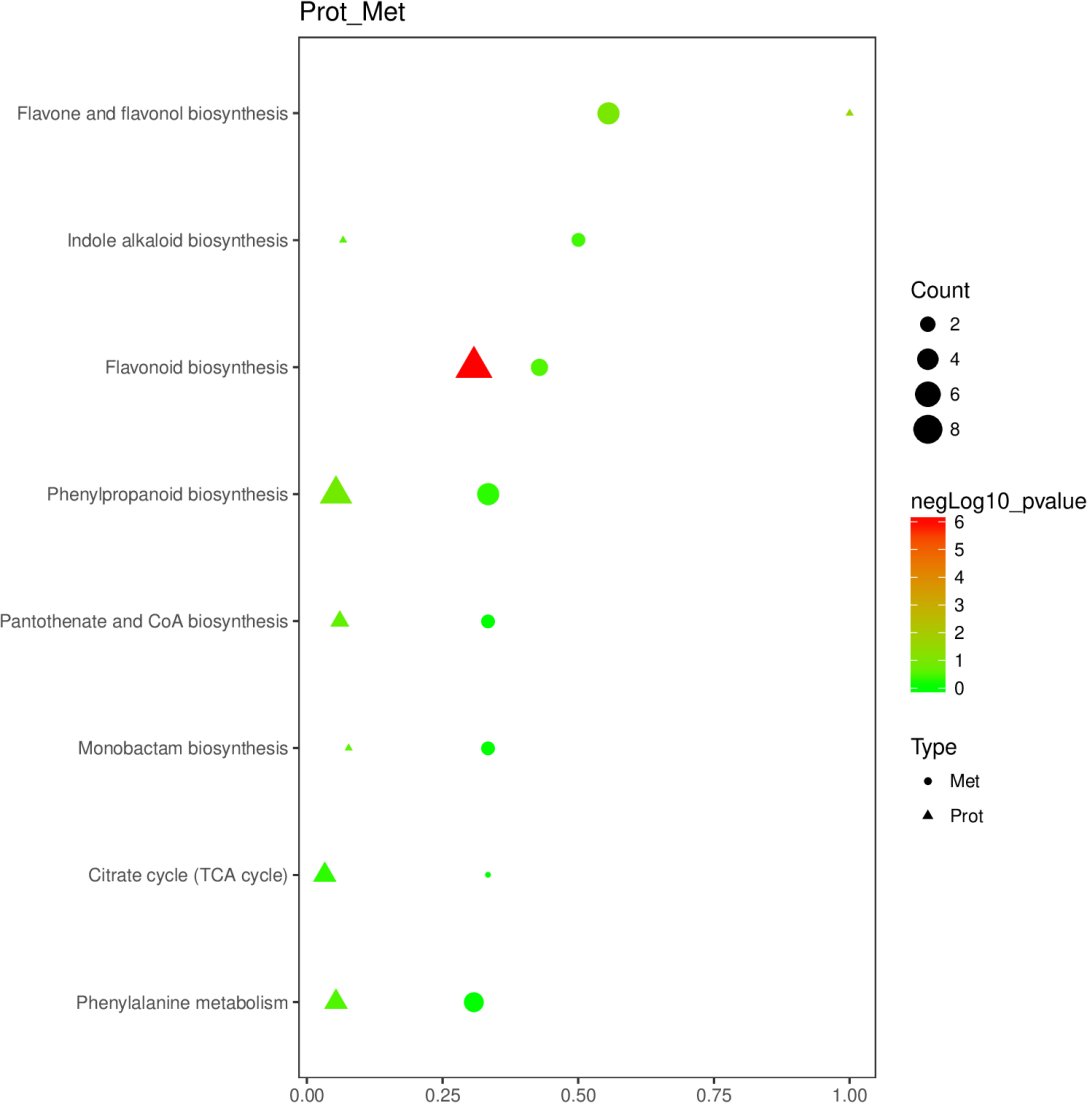
Bubble diagram of enrichment pathway of DEPs and DEMs of cigar tobacco leaves in DDW1 and DDW2 groups. Protein was represented by triangles; metabolite was represented by circles. Horizontal axis indicates the enrichment degree of DEPs and DEMs. Point color represents the P-value, and point size indicates the number of DEPs and DEMs in the corresponding pathway.

Eight primary pathways were identified through KEGG enrichment analysis of the DEPs and DEMs previously screened (**Figure 5**). These pathways included ‘flavonoid biosynthesis’, ‘flavone and flavonol biosynthesis’, ‘indole alkaloid biosynthesis’, ‘phenylpropanoid biosynthesis’, ‘phenylalanine metabolism’, ‘citrate cycle (TCA cycle)’, ‘monobactam biosynthesis’, and ‘pantothenate and CoA biosynthesis’.

**Figure 6:**
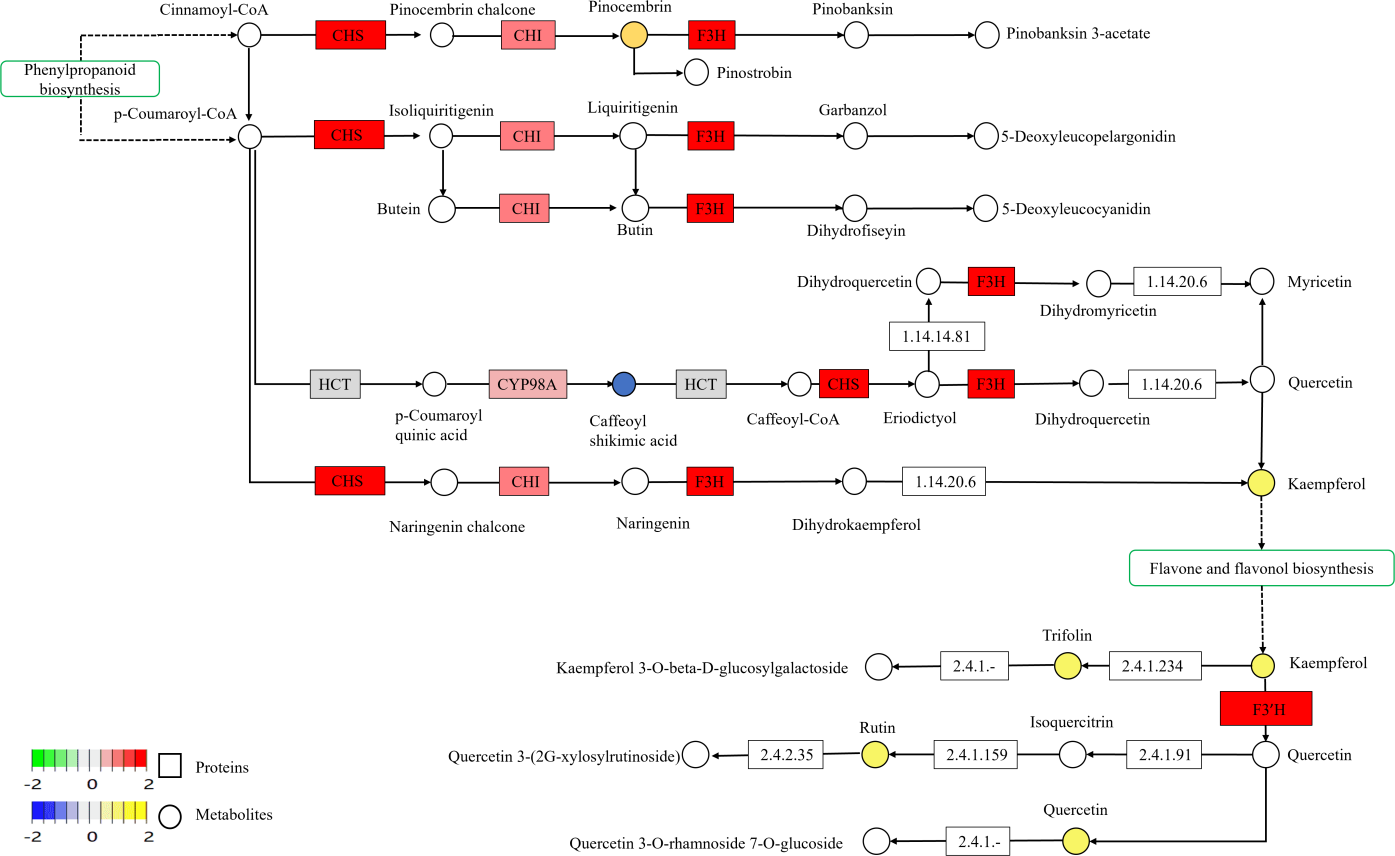
Changes in flavonoid biosynthesis proteins and metabolism. The pathways were drawn based on the KEGG database. CHS, chalcone synthase [EC:2.3.1.74]; CHI, chalcone isomerase [EC:5.5.1.6]; F3H, naringenin 3-dioxygenase [EC:1.14.11.9]; HCT, shikimate O-hydroxycinnamoyltransferase [EC:2.3.1.133]; CYP98A, 5-O-(4-coumaroyl)-D-quinate 3’-monooxygenase [EC:1.14.14.96]; F3’H flavonoid 3’-monooxygenase; flavonoid [EC:1.14.14.82].

Differential proteins and metabolites were analyzed using KEGG pathway mapper tools, with a particular focus on their role in the flavonoid biosynthesis pathway, as shown in **Figure 6**. Within this pathway, three notable differential metabolites were identified: pinocembrin, kaempferol, and caffeoyl shikimic acid. Pinocembrin and kaempferol were up-regulated, whereas caffeoyl shikimic acid was down-regulated. Additionally, several enzymes in this pathway, such as chalcone synthase (CHS), chalcone isomerase (CHI), naringenin 3-dioxygenase (F3H), and 5-O-(4-coumaroyl)-D-quinate 3’-monooxygenase (CYP98A), were predominantly up-regulated, suggesting an overall increase in the activity of flavonoid biosynthesis pathway.

Furthermore, four key differential metabolites-kaempferol, trifolin, rutin, and quercetin-were also up-regulated. Enzymes such as flavonoid 3’-monooxygenase (F3’H) were found to be predominantly up-regulated, indicating enhanced activity in the flavonoid biosynthesis pathway. The interactions and regulatory patterns of these enzymes and metabolites are detailed in **Figure 6**.

## 4 Discussion

Currently, most studies on topping focus primarily on tobacco leaf quality and alkaloid concentrations, often overlooking the mechanical damage it inflicts on the plant. Additionally, these studies typically employ physiological or single-omics methods to explain the process. Incorporating an integrated data approach—encompassing genetics, epigenetics, transcriptomics, and proteomics—can offer a more comprehensive view of plant responses to various abiotic stresses. Liu et al. (2016) mentioned that protein levels do not always reflect metabolite abundance, suggesting that relying solely on one method, like proteomics or metabolomics, may lead to incomplete or inaccurate interpretations. Zapalska-Sozoniuk et al. (2019) stressed the importance of integrating multiple omics approaches for accurate interpretation. By applying multi-omics studies to understand how tobacco plants respond to the abiotic stress (mechanical damage) of topping, we can gain a deeper understanding of their tolerance mechanisms.

Topping in plants results in mechanical damage that disrupts oxidative balance, leading to an increase in ROS (Das and Roychoudhury, 2014). Stress conditions exacerbate this imbalance, causing ROS accumulation that can damage proteins, lipids, and DNA (Hernandez et al., 2009). In response, plants produce various antioxidant enzymes, such as SOD and ascorbate peroxidase (APX), and small molecules like ascorbic acid (AA) and glutathione (GSH) to counteract ROS effects (Mullineaux and Rausch, 2005; Gill and Tuteja, 2010; Miller et al., 2010). Consistent with these results, our study found that the DDW2 group had higher levels of superoxide anion, SOD activity, and MDA content compared to the DDW1 group (**Figures 1**). GO enrichment analysis of DEPs showed a focus on oxidative stress-related terms, such as “flavonoid biosynthesis” and “flavonol and flavone biosynthesis” (**Figure 2C**). Similarly, DEMs were enriched in terms linked to oxidative stress, including “zeatin biosynthesis,” “flavonoid biosynthesis,” and “flavonol and flavone biosynthesis” (**Figure 3**). These findings denote that many metabolic pathways, especially those involved in flavonoid and flavonol/flavone biosynthesis, are altered under topping conditions, indicating a potential link between increased ROS production and the topping process.

KEGG enrichment analysis of DEPs and DEMs revealed the response pathways in tobacco under topping-induced stress. The findings showed that topping conditions significantly alter several metabolic pathways, especially those involved in flavonoid and flavonol/flavone biosynthesis (**Figures 2C**, **3D**, **4**). This implies that flavonoid compounds may play a key role in tobacco’s response to topping (mechanical damage). Flavonoids, a major class of polyphenolic secondary metabolites with over 8000 identified compounds in plants, including flavone, flavonol, and isoflavone, exhibit a range of biological activities due to their unique chemical structures (Yuan et al., 2015). These activities include photoprotection (Harvaux and Kloppstech, 2001), ROS scavenging (Gayomba and Muday, 2020), regulation of auxin transport, pollinator attraction (Kellenberger et al., 2019), pathogen resistance (Yang et al., 2021), stomatal aperture regulation (Watkins et al., 2014), promotion of pollen tube growth (Muhlemann et al., 2018), and influence on root development (Silva-Navas et al., 2016). While most research on flavonoids in tobacco has concentrated on color and quality changes, their role in the plant’s resistance to adverse conditions has been less explored.

In flavonoid biosynthesis, CHS is the initial key enzyme that shifts the metabolic process from phenylpropanoid to flavonoid synthesis (Wang et al., 2017), by catalyzing the reaction between coumaroyl-CoA and malonyl-CoA to generate flavonoid precursors. CHI, the second essential enzyme in this pathway, facilitates the intramolecular cyclization of chalcones to produce flavanones, which are then further modified into various flavonoid structures (Wu et al., 2018; Wang et al., 2017). Studies indicate that both CHS and CHI play significant roles in synthesizing various defensive compounds within the phenylpropanoid pathway and are directly involved in these processes. Plant responses to external stimuli, such as stress and pathogen attacks, involve the rapid activation of CHS, which boosts stress resistance (Turnbull et al., 2004; Petrussa et al., 2013). For instance, incorporating the CHS gene into poplar trees decreased their sensitivity to low temperatures. Similarly, co-expressing CHS and flavonol synthase genes in tomatoes enhanced flavonol production and antioxidant capacity (Verhoeyen et al., 2002). Additionally, overexpression of CHS, CHI, and DFR genes in potato tubers increased anthocyanin and flavonoid levels, thereby improving antioxidant properties (Lukaszewicz et al., 2004). In our study, we observed an upregulation of CHS and CHI in the biosynthesis pathway (**Figure 6**), leading to higher pinosylvin levels. Pinosylvin, a key flavonoid found in propolis with significant antimicrobial properties (Rasul et al., 2013), appears to be linked to the mechanical damage caused by topping, and its increased production could enhance antibacterial effects.

Flavanone 3-hydroxylase (F3H) is a crucial enzyme in the flavonoid biosynthetic pathway, responsible for converting flavanones into dihydroflavonols, which are precursors for flavonol and anthocyanins (Cheng et al., 2013). Regulation of the F3H gene influences plant pigment levels, which enhances plant adaptability and survival, such as increasing resistance to ultraviolet radiation (Prochazkova et al., 2011). It plays an important role in plant morphogenesis, physiological and biochemical functions, and coping with environmental stress (Prochazkova et al., 2011; Ma et al., 2020). The F3H gene has been cloned and identified in several plant species, including *Malus pumila* Mill (Davies, 1993), *Vitis vinifera* L (Sparvoli et al., 1994), *Zea mays* L (Deboer et al., 2009), *Arabidopsis thaliana* (Pelletier and Shirley, 1996), *Glycine max* (Zabala and Vodkin, 2005) and *Carthamus tinctorius* L (Tu et al., 2016). Evidence indicates that F3H is influenced by various environmental factors. For example, gibberellic acid and sucrose boost the expression of EsF3H in epimedium, enhancing the accumulation of flavonoid bioactive compounds (Zeng et al., 2013). Similarly, JA and abscisic acid (ABA) regulate the expression of AtF3H involved in flavonoid biosynthesis in *Arabidopsis* (Loreti et al., 2008; Lewis et al., 2011). Overexpression of F3H in *Arabidopsis* increases resistance to salt and oxidative stress (Li et al., 2017). Our research found that the upregulation of CHS, CHI, and F3H proteins in the flavonoid biosynthesis pathway led to higher levels of dihydroflavonols and subsequent naringenin metabolites. Naringenin, a key flavonoid with antioxidant and anti-inflammatory properties, inhibits protein kinases. Our research shows that CHS and CHI enzymes are mainly involved in synthesizing dihydroflavonols, which, together with FL3, are essential for naringenin production. Naringenin effectively prevents lipid peroxidation and scavenges superoxide anions, aligning with previous studies (Sparvoli et al., 1994). Additionally, increased sunlight and UV-B radiation significantly impact the content and ratio of naringenin and other flavonoids in plant leaves, affecting their photoprotective abilities (Ryan et al., 2002; Zhao et al., 2020; Zhang et al., 2023b).

Flavone and flavonol are classes of natural compounds produced via the phenylpropanoid pathway (Gao et al., 2021). This process begins with phenylalanine, which is first converted into cinnamic acid by phenol oxidase. Cinnamic acid is then converted into coumaric acid through a series of steps. Coumaric acid, a type of organic acid, is subsequently transformed into chalcones through the action of chalcone synthase. Finally, chalcones are converted into flavone or flavonol by flavon synthase (Wu et al., 2018).

Flavonoid 3’-hydroxylase (F3’H, EC: 1.14.13.21) is a cytochrome P450 monooxygenase enzyme (Shi et al., 2006; Li et al., 2021) belonging to the CYP75 subfamily and plays a crucial role in the synthesis of flavonoid compounds. Our study found that topping increases F3’H expression. This enzyme acts on various substrates, including apigenin, naringenin, kaempferol, and dihydrokaempferol. These results align with previous findings that F3’H is upregulated under stress conditions, boosting resistance. For instance, in *Citrus sinensis*, the gene CsF3’H is significantly induced by drought stress. In transgenic *Arabidopsis* plants overexpressing CsF3’H, there are lower levels of ROS and higher levels of antioxidant flavonoids and antioxidant enzyme activity compared to wild-type plants, thereby improving drought resistance (Guo et al., 2011).

It was also found that trifolin, rutin, and quercetin experienced alterations in their biosynthesis pathways. Following topping, levels of these compounds increased, implying their antioxidant properties. Liu et al. (2021) investigated lilac (*Syringa*) responses to light stress and discovered that light can regulate the expression of key genes in the rutin synthesis pathway, such as 4CL1, CYP73A, and CYP75B1, leading to higher rutin levels. Conversely, salt stress impaired germination and seedling growth in *Apocynum venetum* L., resulting in reduced total flavonoid content. However, the content of the flavonol quercetin increased, with the upregulation of genes involved in its synthesis, including AvF3’H, AvF3H, and AvFLS (Xu et al., 2020).

Research has demonstrated that the biosynthesis of flavonoids, flavones, and flavonols can boost a plant’s antioxidant defense (Prochazkova et al., 2011; Petrussa et al., 2013). This study found that topping significantly influenced the biosynthesis of these compounds, along with the activities of O_2_^-^, SOD, and MDA. Protein and metabolism analysis revealed that CHS, CHI, F3H, and F3’H were upregulated, and the levels of metabolites such as kaempferol, clover, rutin, and quercetin were increased. These changes support the accumulation of antioxidant enzymes, help maintain oxidative balance, and protect against oxidative stress by continuously synthesizing and accumulating these compounds. They also shield lipids and membrane proteins from oxidative damage, neutralize ROS, and reduce oxidative cell damage. While these findings shed light on the activation of antioxidant mechanisms following topping in cigar tobacco, further research is needed to gain a more comprehensive understanding of the underlying protein and metabolic processes.

## 5 Conclusion

In this study, we revealed that topping significantly affects the intrinsic proteins and metabolites in cigar tobacco leaves. This impairment modifies the amount of proteins associated with flavonoid biosynthesis and flavone and flavonol biosynthesis, including CHS, CHI, F3H, and F3’H, as well as the accumulation of the metabolic products kaempferol, trifolin, rutin, and quercetin. Consequently, this encourages the buildup of antioxidative enzymes. To maintain oxidative balance and counteract oxidative stress, flavonoids are continuously produced and accumulated. This process helps protect lipids and membrane proteins from oxidative damage, neutralizes ROS, and impedes their formation, thus mitigating oxidative cell damage. Our findings offer novel insights into the metabolomic and proteomic responses of cigar tobacco to topping.

## Data Availability

The original contributions presented in the study are publicly available. This data can be found here: ProteomeXchange, PXD060658.
