# Supplementary material for: Proteomic and metabolomic insights into the impact of topping treatment on cigar tobacco
Source: Front Plant Sci. 2025 Feb 20;15:1425154. doi: 10.3389/fpls.2024.1425154 (PMC11882365; doi:10.3389/fpls.2024.1425154)
Supplement: Supplementary file 1 [file DataSheet1.pdf]

Proteomic and Metabolomic Insights into the Impact of Topping Treatment on Cigar Tobacco

Dong Guo†, Huajun Gao†, Tongjing Yan, Changjian Xia, Beisen Lin, Xiaohua Xiang,

Bin Cai*, Zhaoliang Geng*

1. Haikou Cigar Research Institute, Hainan Province Company, China National Tobacco Corporation, Haikou 571100, China

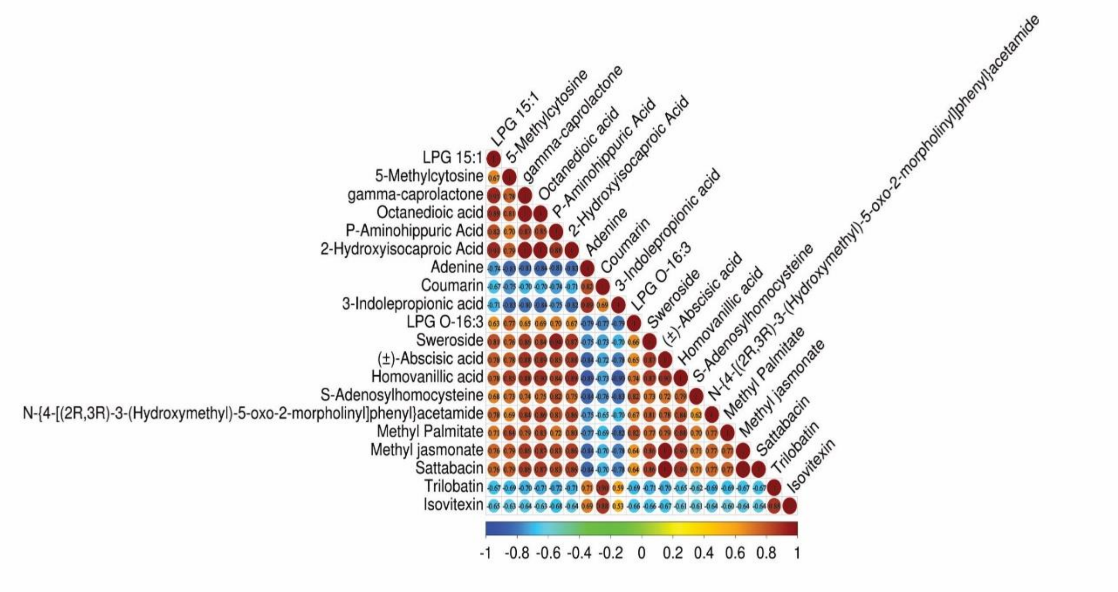

Figure S1. Correlation analysis shows that

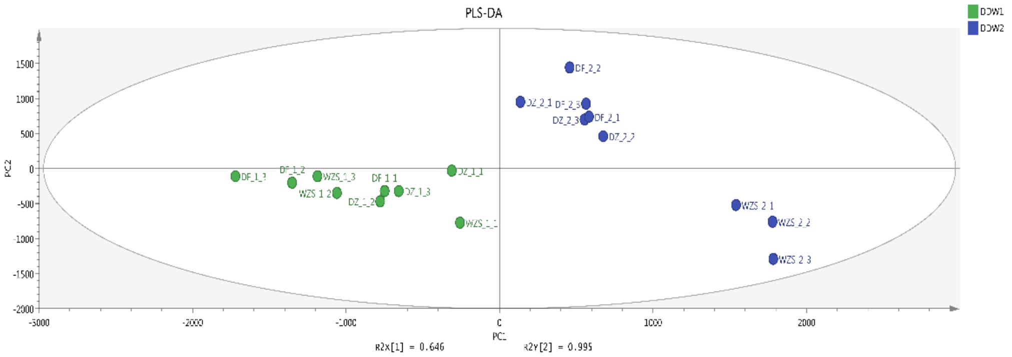

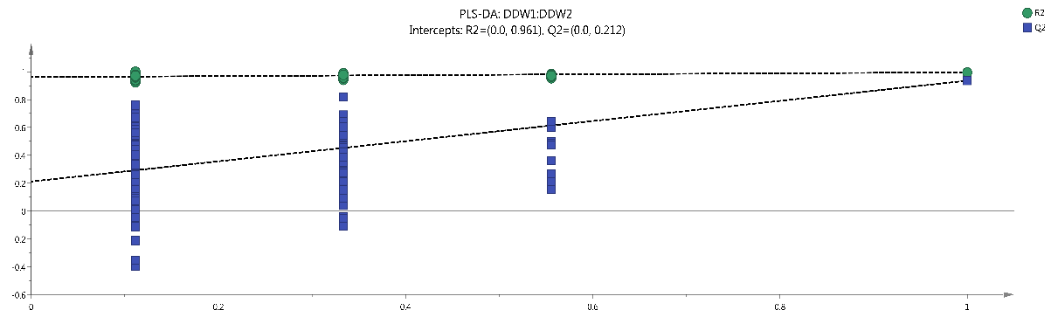

Figure S2. Identification of Proteins. PLS-DA score scatter diagram and sequencing verification diagram of cigar tobacco leaves in DDW1 and DDW2 groups.

Cluster	color	Pathway
1	Red	Porphyrin and chlorophyll metabolism; Selenocompound metabolism; Amino sugar and nucleotide sugar metabolism; Biosynthesis of secondary metabolites; Metabolic pathways
2	Green	Phenylalanine, tyrosine and tryptophan biosynthesis; Flavonoid biosynthesis; Glyoxylate and dicarboxylate metabolism; Biosynthesis of amino acids; Pyruvate metabolism
3	blue	Flavone and flavonol biosynthesis; Flavonoid biosynthesis; Steroid biosynthesis; Biosynthesis of secondary metabolites

Table S1 The PPI analysis of differentially expressed protein.
This table details the main functional classification of each cluster.

Table S2 Differentially expressed proteins identification of seedling tobacco DDW1 and DDW2

Protein	FC	Pvalue	log2FC
A0A059TCM2	0.447178314	3.95935E-05	-1.161077867
A0A059XID0	2.457145615	3.56882E-05	1.296983357
A0A075EYT9	2.309809019	0.041043713	1.207773571
A0A075EZT1	0.423879253	0.011824355	-1.238274741
A0A075F1U5	2.071577201	7.02586E-07	1.050729586
A0A097BU08	0.139396801	0.000947253	-2.842730641
A0A0A8WHC1	0.432229651	1.59177E-05	-1.21013005
A0A0A8WJH5	2.470918699	0.006516304	1.305047542
A0A0A8WK76	0.436930975	3.21666E-08	-1.194522709
A0A0C4Y311	3.090597759	3.49108E-06	1.6278859
A0A0F7R4E4	2.029741365	4.94486E-06	1.021295907
A0A0N7CSC8	0.413794227	9.52512E-05	-1.273014577
A0A1S3WXX8	0.425651711	0.005140206	-1.232254666
A0A1S3WZN5	0.488627432	0.0002961	-1.033193235
A0A1S3X059	0.463947858	0.03005326	-1.107965423
A0A1S3X0G8	2.091649273	0.003049832	1.064640961

A0A1S3X0I2	2.767682919	0.002438201	1.468678669
A0A1S3X0J2	0.487849675	1.81874E-08	-1.035491429
A0A1S3X1G1	2.67816377	0.001388734	1.421244184
A0A1S3X1L8	0.45972096	0.000952921	-1.12116965
A0A1S3X1Q3	2.693515747	0.000828211	1.4294905
A0A1S3X200	0.432635873	0.004908005	-1.208774799
A0A1S3X233	2.111162576	0.000770231	1.078037682
A0A1S3X4I9	2.125348413	5.35672E-05	1.087699365
A0A1S3X4K3	0.427515177	0.005053679	-1.225952457
A0A1S3X4M2	0.181666245	0.023045452	-2.460637714
A0A1S3X4U8	2.047928255	2.55999E-09	1.034165175
A0A1S3X501	2.222412198	0.035421582	1.152126423
A0A1S3X5H9	2.263444029	6.21167E-06	1.178519632
A0A1S3X5W6	2.001572763	7.67302E-05	1.001134063
A0A1S3X5W8	0.469869352	0.0002288	-1.089668427
A0A1S3X699	2.101121521	5.13969E-05	1.071159605
A0A1S3X7H4	2.112573841	0.004534229	1.079001769
A0A1S3X7T6	0.424413316	0.014873991	-1.236458175
A0A1S3X7Y0	2.237226313	3.44871E-06	1.161711204
A0A1S3X936	2.099685939	0.002718701	1.070173552
A0A1S3X969	0.307223613	0.00100272	-1.702638988
A0A1S3X9M1	2.041683528	0.000630336	1.029759258
A0A1S3XA86	2.015455042	0.002174728	1.011105602
A0A1S3XAU0	2.033990123	3.34944E-05	1.024312674
A0A1S3XBH0	2.918025971	4.3654E-05	1.544992724
A0A1S3XBT0	3.235416838	0.033431808	1.693951595
A0A1S3XC04	2.393097076	2.04054E-06	1.258878921
A0A1S3XC15	2.053401604	0.000567608	1.038015817
A0A1S3XC51	2.217650203	5.74169E-05	1.149031822
A0A1S3XC54	2.392676728	0.01708378	1.258625489
A0A1S3XCD2	2.387101649	3.2457E-05	1.255260001
A0A1S3XEV7	2.152548816	1.76457E-05	1.106045956
A0A1S3XFL7	2.38743573	4.82143E-08	1.255461896
A0A1S3XG66	2.312136099	0.014406938	1.209226322
A0A1S3XGJ1	0.472696056	0.013904065	-1.081015266
A0A1S3XHA2	0.490412014	0.010907196	-1.027933773
A0A1S3XHQ5	0.298992661	0.015953287	-1.741818022
A0A1S3XJG1	0.353470482	0.001578596	-1.500338352
A0A1S3XKG1	0.395831597	0.0422601	-1.337041315
A0A1S3XLY5	2.230644086	0.000844551	1.15746034
A0A1S3XMX2	3.843954805	2.20471E-06	1.942591374
A0A1S3XP26	3.472189269	5.54611E-05	1.795845591
A0A1S3XQ01	0.454690655	3.70969E-07	-1.137042741
A0A1S3XSC6	2.825921474	6.01321E-08	1.498721377

A0A1S3XT40	0.35993097	0.00441829	-1.474207851
A0A1S3XTF0	2.493892465	9.9937E-05	1.318399258
A0A1S3XTH4	0.447099666	0.022857476	-1.161331626
A0A1S3XTH9	3.241614322	3.56696E-06	1.696712453
A0A1S3XTT0	2.217422949	2.05174E-06	1.148883974
A0A1S3XV79	0.483114455	0.030685551	-1.049563077
A0A1S3XXM2	0.337385707	0.046252788	-1.567529239
A0A1S3XXX1	0.495917421	6.75211E-06	-1.011828187
A0A1S3XY03	0.344971781	1.40056E-06	-1.535449744
A0A1S3XY22	3.397076636	4.03636E-05	1.764293765
A0A1S3XYI2	2.369665314	2.30143E-05	1.244683311
A0A1S3XYP9	2.205954347	0.000648985	1.141402934
A0A1S3Y0V1	2.638955135	1.96797E-06	1.399966823
A0A1S3Y185	2.453425315	2.65515E-06	1.294797355
A0A1S3Y1G9	2.876578601	0.000731304	1.524353891
A0A1S3Y2G8	0.397126542	3.24518E-05	-1.332329308
A0A1S3Y2X5	2.920539579	0.002117828	1.546234936
A0A1S3Y357	0.445018414	0.008160056	-1.168063061
A0A1S3Y3L0	0.464470701	9.20223E-05	-1.106340501
A0A1S3Y4L5	0.485994034	0.006989517	-1.040989491
A0A1S3Y546	0.452824478	0.000711412	-1.142976149
A0A1S3Y5C0	2.478054646	1.08684E-07	1.309208002
A0A1S3Y635	2.726781767	1.66781E-05	1.447199241
A0A1S3Y729	2.160519637	0.000873033	1.111378344
A0A1S3Y788	2.119066647	0.030916543	1.083428963
A0A1S3Y7P7	0.460576908	0.023746902	-1.118486016
A0A1S3Y851	2.106929919	6.03505E-07	1.075142328
A0A1S3Y8E2	2.213680402	0.000696263	1.146446949
A0A1S3Y8F0	2.127285593	1.50003E-05	1.089013731
A0A1S3Y8L4	0.404431014	3.7053E-08	-1.306034461
A0A1S3Y976	0.491778087	0.000100317	-1.023920644
A0A1S3Y9Q0	0.463668234	0.003669921	-1.108835203
A0A1S3YB24	2.857699359	0.002607476	1.514854147
A0A1S3YCM0	0.317414001	0.022186587	-1.655562328
A0A1S3YCS2	2.3190768	1.20976E-05	1.213550598
A0A1S3YD65	0.470338452	0.00429044	-1.088228811
A0A1S3YEK6	2.177675001	3.40812E-05	1.122788661
A0A1S3YEX4	2.498387019	0.020177654	1.320996979
A0A1S3YFE9	2.014834457	1.64203E-06	1.010661309
A0A1S3YFW3	0.314480884	0.002345024	-1.66895577
A0A1S3YHC5	0.484933301	0.002481967	-1.044141767
A0A1S3YLB2	2.583467176	9.9488E-08	1.369308554
A0A1S3YLB4	2.21375255	0.000313428	1.146493969
A0A1S3YLS5	0.410961697	0.033718278	-1.282924158

A0A1S3YMA4	2.348295819	8.57601E-05	1.231614159
A0A1S3YMI7	0.459658921	0.048493863	-1.121364355
A0A1S3YN76	0.422694251	0.044282034	-1.242313606
A0A1S3YP51	0.459053543	0.001015282	-1.12326566
A0A1S3YPQ0	0.280033487	8.12851E-05	-1.836328737
A0A1S3YQV4	2.002129425	0.001697607	1.001535238
A0A1S3YR69	2.15760634	0.000192243	1.109431666
A0A1S3YT22	2.061314068	0.007420043	1.043564335
A0A1S3YTT1	2.89423543	0.000176937	1.533182282
A0A1S3YU74	2.767944961	3.47925E-07	1.468815256
A0A1S3YU89	3.25964617	0.001213846	1.70471537
A0A1S3YVY6	2.020412935	2.11254E-05	1.014650183
A0A1S3YW23	0.444195814	0.00066168	-1.170732296
A0A1S3YW44	2.267803107	0.001943963	1.18129539
A0A1S3YW49	2.134464915	0.031547171	1.093874449
A0A1S3YW96	2.281240539	0.006691342	1.189818575
A0A1S3YXD0	0.42591637	0.000968925	-1.231357916
A0A1S3YYQ0	0.291185273	0.000455344	-1.779990704
A0A1S3Z084	0.442925343	2.95755E-05	-1.174864547
A0A1S3Z0J0	2.424650859	0.024763833	1.277777019
A0A1S3Z0R2	2.454890926	1.43931E-06	1.295658925
A0A1S3Z2I2	2.018583465	1.6082E-06	1.013343241
A0A1S3Z2I3	2.093808362	0.00077341	1.066129404
A0A1S3Z327	2.924907445	8.76394E-06	1.548390973
A0A1S3Z3G4	2.90608593	3.94446E-06	1.539077363
A0A1S3Z4G5	0.402357621	3.74194E-05	-1.313449736
A0A1S3Z4S7	2.034775018	3.46491E-05	1.024869286
A0A1S3Z4T2	0.308628756	0.039182502	-1.696055607
A0A1S3Z4T3	0.416568793	0.013297887	-1.263373331
A0A1S3Z4V1	0.486830462	0.037282357	-1.038508651
A0A1S3Z500	0.447304719	0.015931589	-1.160670117
A0A1S3Z5N4	2.084819896	6.61147E-05	1.059922757
A0A1S3Z5V1	2.669471766	0.006115434	1.41655429
A0A1S3Z7D3	0.371466274	1.22105E-06	-1.428696862
A0A1S3Z7X6	0.226938733	0.034130657	-2.139625234
A0A1S3Z8F3	0.314216054	0.002278852	-1.6701712
A0A1S3Z968	2.15962541	0.020109219	1.110781097
A0A1S3Z9M9	2.055714195	0.013053587	1.039639701
A0A1S3ZA12	2.290771624	6.85324E-05	1.195833638
A0A1S3ZBB2	0.459887468	0.018101123	-1.120647211
A0A1S3ZBV1	0.469868257	0.036046802	-1.089671788
A0A1S3ZC29	0.499963169	0.024024524	-1.000106277
A0A1S3ZDB8	2.90212851	8.88111E-07	1.537111405
A0A1S3ZDU0	0.347986825	0.006422869	-1.522895407

A0A1S3ZEL4	2.290718966	0.00126188	1.195800474
A0A1S3ZEW7	0.295088769	4.00774E-05	-1.760779081
A0A1S3ZEZ8	0.30149574	8.6884E-05	-1.729790479
A0A1S3ZFC4	2.078335402	3.06406E-06	1.055428495
A0A1S3ZGC4	0.231370899	0.00017266	-2.111720675
A0A1S3ZGP8	2.698054708	0.014554106	1.431919602
A0A1S3ZHQ4	2.587463269	0.006995257	1.371538383
A0A1S3ZHV1	2.172728388	2.81891E-06	1.119507835
A0A1S3ZI99	2.25890488	5.69893E-05	1.175623522
A0A1S3ZIK6	2.453736842	3.71213E-07	1.294980531
A0A1S3ZJS9	3.788070572	4.00169E-06	1.921463209
A0A1S3ZK19	2.227507773	0.000342275	1.155430466
A0A1S3ZK57	0.351715951	0.002145875	-1.50751733
A0A1S3ZK93	2.204886162	0.034896164	1.140704171
A0A1S3ZKC4	2.135381371	2.90435E-06	1.094493753
A0A1S3ZKM4	0.42918134	9.87117E-06	-1.220340743
A0A1S3ZKM7	0.288559796	0.000636184	-1.793057787
A0A1S3ZKV6	2.230471481	0.000279749	1.157348702
A0A1S3ZL13	2.602010892	0.048752615	1.379627001
A0A1S3ZL81	0.275087346	0.021645706	-1.862038315
A0A1S3ZLJ6	0.489265531	0.004159357	-1.031310447
A0A1S3ZLZ7	2.021024436	7.41874E-07	1.015086765
A0A1S3ZMG5	2.381154409	0.021259379	1.251661177
A0A1S3ZMQ1	2.416451546	8.19414E-05	1.272890067
A0A1S3ZNA2	2.313200627	0.000103829	1.209890399
A0A1S3ZNH5	2.439867494	4.12094E-05	1.286802799
A0A1S3ZNN0	2.707887336	0.035224298	1.437167716
A0A1S3ZPA1	2.20127168	0.001250964	1.138337213
A0A1S3ZPA7	2.233255267	2.7049E-05	1.159148164
A0A1S3ZPC5	2.034558486	0.000585843	1.024715753
A0A1S3ZPC7	2.197732863	0.007884115	1.136016036
A0A1S3ZR33	2.129113128	5.12395E-05	1.090252608
A0A1S3ZR63	0.465504425	0.044852319	-1.103133213
A0A1S3ZRI7	0.422621246	0.016066743	-1.2425628
A0A1S3ZRV5	2.378770038	1.61256E-07	1.250215809
A0A1S3ZSG6	2.340038085	4.78424E-07	1.226532011
A0A1S3ZVF0	2.12433257	0.004889253	1.087009642
A0A1S3ZW98	0.479239379	0.047829315	-1.061181635
A0A1S3ZWB9	2.354119284	2.07853E-05	1.235187424
A0A1S3ZXH6	2.014893736	0.000144171	1.010703754
A0A1S3ZXT9	0.437519629	0.004008875	-1.192580352
A0A1S3ZYP6	3.199205609	5.05448E-05	1.677713715
A0A1S3ZZ32	2.232761176	0.024735229	1.158828944
A0A1S4A008	3.160455553	0.00013984	1.660132526

A0A1S4A0N3	2.655868763	9.58675E-05	1.409183859
A0A1S4A0P8	2.004646636	3.55603E-05	1.003347952
A0A1S4A107	0.473662128	7.67918E-05	-1.07806977
A0A1S4A1E8	2.009498833	0.00156002	1.00683574
A0A1S4A2H1	2.379484063	0.000170322	1.250648792
A0A1S4A2T4	0.477266769	0.000496225	-1.067132205
A0A1S4A3B3	2.070873766	9.10074E-06	1.050239614
A0A1S4A5R2	3.371131277	0.000206501	1.753232809
A0A1S4A5Y7	2.140777132	0.013106019	1.09813461
A0A1S4A648	2.0579881	0.011312729	1.04123464
A0A1S4A687	0.470556107	5.21486E-05	-1.08756134
A0A1S4A764	0.464829116	0.039073103	-1.105227655
A0A1S4A7I0	0.378826668	0.002745637	-1.400390201
A0A1S4A899	2.209890934	6.91159E-08	1.143975169
A0A1S4A9S4	2.039020586	0.002636108	1.027876341
A0A1S4A9Y6	2.012243525	5.65141E-07	1.008804913
A0A1S4AAA5	0.465497686	0.00143295	-1.103154099
A0A1S4AAB4	0.424143767	0.002304677	-1.237374732
A0A1S4AB06	2.506287226	0.004371466	1.32555176
A0A1S4ABD2	0.356324161	0.000836235	-1.488737785
A0A1S4ABX7	2.061848849	0.01566733	1.043938575
A0A1S4ADR4	2.406011048	0.02671212	1.266643267
A0A1S4AEH4	2.141905332	7.20992E-05	1.098894717
A0A1S4AER7	0.237159395	0.002815368	-2.076071075
A0A1S4AGB5	2.21552624	8.796E-06	1.147649414
A0A1S4AGR0	0.47861169	0.003393597	-1.063072461
A0A1S4AI83	2.01205009	4.97726E-05	1.008666222
A0A1S4AJ04	0.447114669	0.003737058	-1.161283215
A0A1S4AJ91	2.119702635	0.000155896	1.083861889
A0A1S4AJA4	0.240468518	6.58543E-06	-2.056080067
A0A1S4AKG4	0.488662202	4.07007E-05	-1.033090579
A0A1S4AL11	0.305722001	0.004086081	-1.709707719
A0A1S4AM80	2.129360678	2.60852E-05	1.090420339
A0A1S4AMX1	0.429567552	0.002394262	-1.219043073
A0A1S4ANA3	0.432019793	0.000243098	-1.210830683
A0A1S4ANJ7	0.445683107	1.10243E-05	-1.165909817
A0A1S4AP67	0.357901413	0.003110043	-1.482365854
A0A1S4APK5	0.433300248	0.020418969	-1.206561031
A0A1S4AQF6	2.436604518	1.58275E-06	1.284872108
A0A1S4ARB9	3.544027876	0.001548161	1.825389952
A0A1S4AT44	2.110871924	0.000129938	1.077839047
A0A1S4ATK0	3.627667222	4.75969E-08	1.859042119
A0A1S4AU23	2.282222765	0.028208634	1.190439618
A0A1S4AUD7	2.200234339	0.000463551	1.137657188

A0A1S4AVG3	2.047028729	9.56611E-06	1.03353135
A0A1S4AW97	0.268600135	2.70439E-07	-1.896468063
A0A1S4AWK3	2.700275339	1.59472E-05	1.433106522
A0A1S4AX59	2.854022105	7.17917E-05	1.512996509
A0A1S4AXA8	0.445980476	0.012413776	-1.164947541
A0A1S4AXH8	2.177740191	0.000103377	1.122831848
A0A1S4AY65	4.538045258	2.64356E-05	2.182070997
A0A1S4B0U5	2.263654923	3.1858E-05	1.178654047
A0A1S4B192	2.080368387	1.24105E-05	1.05683902
A0A1S4B1G1	3.457338541	1.92195E-06	1.789661878
A0A1S4B1M3	2.662624313	0.001750791	1.412848884
A0A1S4B264	2.463427624	3.66539E-07	1.300667085
A0A1S4B2F2	2.266038495	1.71159E-06	1.180172369
A0A1S4B2P7	2.12059299	1.73306E-05	1.084467748
A0A1S4B3D7	2.143679735	9.37958E-06	1.100089384
A0A1S4B3S7	3.402717315	1.59634E-06	1.766687302
A0A1S4B3X5	2.472523486	0.001894443	1.305984225
A0A1S4B413	2.310128275	0.002764484	1.207972962
A0A1S4B436	0.483583616	0.036118446	-1.048162729
A0A1S4B514	0.4903998	0.001967069	-1.027969704
A0A1S4B6R7	2.216442287	0.000762228	1.148245797
A0A1S4B7D8	0.492864029	0.006788977	-1.020738403
A0A1S4B7K4	0.483988362	9.57321E-07	-1.046955737
A0A1S4B8F5	0.475122319	0.004821076	-1.073629115
A0A1S4B8J4	2.024848693	1.04519E-05	1.017814107
A0A1S4BAY8	2.810584922	0.002536511	1.490870406
A0A1S4BBB5	2.127383875	1.58882E-05	1.089080384
A0A1S4BCN3	2.111679514	0.001149913	1.078390896
A0A1S4BEN9	2.180048765	0.002867217	1.124360407
A0A1S4BFM2	0.446429446	0.009300122	-1.163495907
A0A1S4BG49	2.166831168	0.000252163	1.115586748
A0A1S4BG93	0.485716647	0.006120468	-1.041813162
A0A1S4BGH7	4.111432232	6.97914E-08	2.03964105
A0A1S4BHB3	0.391187895	0.033376335	-1.354066366
A0A1S4BHD3	2.113939057	0.001759391	1.079933786
A0A1S4BIA9	0.393446943	0.008103843	-1.345758996
A0A1S4BIC7	2.31687269	1.18063E-05	1.212178772
A0A1S4BM26	4.205524432	0.002068125	2.072285717
A0A1S4BNB5	2.250697084	6.79944E-06	1.170371901
A0A1S4BNE4	0.446563973	0.003796048	-1.163061231
A0A1S4BNP5	0.447384089	1.4203E-05	-1.160414145
A0A1S4BNX3	0.213652972	0.046909946	-2.226658709
A0A1S4BPF8	2.005443816	0.000102328	1.003921548
A0A1S4BPW2	2.18057917	8.02458E-08	1.124711371

A0A1S4BQ42	0.16531844	0.008217653	-2.596680436
A0A1S4BQF9	0.34860798	0.002284642	-1.5203225
A0A1S4BRZ6	0.380944753	1.7004E-07	-1.392346312
A0A1S4BS12	4.337135131	0.000142576	2.116742394
A0A1S4BT05	2.035969801	0.000877285	1.025716163
A0A1S4BUP5	0.312104731	0.004097061	-1.679897871
A0A1S4BUQ3	2.443138489	0.001079105	1.288735645
A0A1S4BV79	0.47476612	0.019991967	-1.074711109
A0A1S4BVI1	0.431994306	0.0079779	-1.2109158
A0A1S4BX50	3.494876045	0.035501748	1.805241288
A0A1S4BXI3	2.019567036	6.7127E-07	1.014046034
A0A1S4BY05	2.05709412	7.11693E-05	1.040607804
A0A1S4BYC7	2.367274147	0.003529857	1.24322679
A0A1S4BYM8	0.350841752	0.03004235	-1.51110765
A0A1S4BZ12	0.472323349	0.002618115	-1.082153239
A0A1S4BZR5	2.223461836	1.14742E-06	1.152807642
A0A1S4BZS0	3.455043535	0.001238981	1.788703889
A0A1S4BZT5	0.3532611	0.044855902	-1.501193204
A0A1S4C003	3.519906377	0.001889581	1.815537056
A0A1S4C0A9	0.364374193	0.006885779	-1.456507312
A0A1S4C0H9	0.498688149	0.008502622	-1.003790176
A0A1S4C0U2	2.003212161	0.000719887	1.002315225
A0A1S4C3J4	2.003705504	0.001187246	1.002670483
A0A1S4C5D0	2.890546082	0.011072191	1.531342073
A0A1S4C661	2.313435816	0.012014112	1.210037073
A0A1S4C9S4	0.2595374	0.026484517	-1.945985647
A0A1S4CAD2	0.268088629	0.02469604	-1.899218064
A0A1S4CAG6	0.48097323	0.002941627	-1.055971496
A0A1S4CBG9	0.403504972	0.00110169	-1.309341646
A0A1S4CD46	0.388614078	0.017871551	-1.36358993
A0A1S4CDQ0	0.271431053	0.031040947	-1.881342313
A0A1S4CF02	2.864665968	6.33022E-05	1.518366925
A0A1S4CFC3	0.457313748	0.014034867	-1.128743805
A0A1S4CHK4	0.08280042	0.039965253	-3.594218098
A0A1S4CHM7	2.803639276	0.001047281	1.48730074
A0A1S4CHV0	2.829256759	3.35598E-06	1.500423109
A0A1S4CIA8	2.204257196	0.001289203	1.14029257
A0A1S4CJP2	2.302858758	0.000787697	1.203425928
A0A1S4CJS8	2.344474967	1.78159E-05	1.229264875
A0A1S4CLM0	2.527022585	0.035982023	1.337438558
A0A1S4CMM1	2.06559699	2.99618E-06	1.046558803
A0A1S4CMM8	0.409498517	0.001798814	-1.288069868
A0A1S4CN15	2.40727529	0.002697835	1.267401134
A0A1S4CPF1	2.076746809	0.022942685	1.054325337

A0A1S4CQ36	6.980124979	0.003525745	2.803252868
A0A1S4CQI1	2.149342262	0.000466513	1.103895237
A0A1S4CQX6	2.365483411	9.05347E-07	1.242135043
A0A1S4CST7	0.34126378	1.95773E-05	-1.551040791
A0A1S4CSW7	0.354051027	2.04807E-05	-1.497970792
A0A1S4CSY2	0.385411532	0.022045328	-1.375528355
A0A1S4CUR3	0.444412373	7.05373E-07	-1.170029112
A0A1S4CW19	0.437258064	0.027610327	-1.193443105
A0A1S4CWD4	0.485374406	0.03261347	-1.042830057
A0A1S4CXR2	0.417621904	0.045223974	-1.259730714
A0A1S4CY94	0.220215714	0.000392797	-2.183010676
A0A1S4CYB6	2.044055567	1.27308E-05	1.031434416
A0A1S4CYY0	3.841574129	0.001386439	1.941697593
A0A1S4D0M8	0.4311023	0.000715739	-1.213897834
A0A1S4D0Q6	2.18526631	0.01704305	1.127809106
A0A1S4D1X6	2.256402741	0.003022394	1.174024594
A0A1S4D1Z6	2.346760866	0.019052502	1.230670839
A0A1S4D3A6	2.170241475	5.08411E-05	1.117855575
A0A1S4D3L8	2.150902993	2.60209E-08	1.10494246
A0A1S4D663	2.697334424	1.05671E-08	1.431534403
A0A1S4D6Y6	2.203419534	2.67753E-06	1.139744212
A0A1S4D8N5	2.011116318	0.00088874	1.007996526
A0A1S4D902	2.020904937	0.005580388	1.015001459
A0A1S4DB92	0.412275933	0.000432189	-1.278317851
A0A1S4DBI8	2.539695843	0.000395455	1.344655728
A0A1S4DBY6	2.198550787	0.004567038	1.13655286
A0A1S4DBZ0	2.516981749	3.83229E-08	1.331694755
A0A1S4DCM1	0.34751101	0.002884752	-1.52486941
A0A1S4DCY7	0.397578123	0.00814058	-1.330689723
A0A1S4DCZ5	0.469780991	0.014146971	-1.089939757
A0A1S4DEH2	0.44183621	0.020644843	-1.178416438
A0A1S4DF33	2.049685637	0.000148486	1.035402659
A0A1S4DFL7	2.273827185	1.71566E-05	1.185122611
A0A1S4DFY0	0.414505161	1.04266E-05	-1.270538031
A0A1S4DGM5	0.409309367	0.001249729	-1.288736412
A0A1S4DIQ9	0.469371101	0.001847621	-1.091199077
A0A1S4DJQ0	2.343057885	0.008436434	1.228392596
A0A1S4DJY3	0.373325398	0.008520513	-1.421494434
A0A1S4DM35	2.855749475	0.000159955	1.513869422
A0A1S4DNM9	3.011900292	1.08474E-05	1.590674011
A0A1S4DPF6	2.549971847	0.000369386	1.350481319
A0A1S4DQI3	2.023155651	8.24718E-05	1.016607318
A0A1S4DQY5	5.086803304	4.35921E-05	2.346759309
A0A1S4DR24	0.183438363	0.009828547	-2.446632709

A0A1S4DRD3	0.477572751	0.00012904	-1.066207572
A0A1S4DRL6	3.455529631	6.71306E-06	1.78890685
A0A1S4DSA8	5.606240016	0.00013794	2.487033511
A1KYB0	2.372327225	0.005447898	1.246303021
A9ZMI9	2.471868855	0.000286295	1.305602203
D2KWM9	0.432571728	0.001714891	-1.208988718
J7MDG9	2.699183266	0.046207858	1.432522935
O24163	2.153900852	0.008048307	1.106951842
O49908	0.456842215	0.018815768	-1.130232123
O49910	0.398261394	0.001297585	-1.328212459
P25317	0.327508575	0.00613923	-1.610395415
P93350	2.269347276	0.000291196	1.1822774
Q04065	2.127822387	5.68331E-05	1.089377732
Q43576	0.344241418	0.000706795	-1.538507406
Q589Y3	0.440486705	0.007173755	-1.182829618
Q9LRC7	0.360554135	0.02514688	-1.471712207
T2BRU8	0.485788923	0.001896583	-1.041598502

Table S3 Information of metabolites analyzed in this study.

Name	Formula	Kegg_ID	HMDB_ID	Lipidmaps_ID	Classify
Eleutheroside E	C34 H46 O18	cpd:C20786	HMDB0036410	NA	Organic oxygen compounds
Ginsenoside Rg3	C42 H72 O13	cpd:C20778	HMDB0039546	LMPR010608001<br>0	Lipids and lipid-like molecules
Ptaquiloside	C20 H30 O8	cpd:C19515	HMDB0242690	NA	Organic oxygen compounds
Prohydrojasmon	C15 H26 O3	cpd:C18538	NA	NA	Lipids and lipid-like molecules
Pheophorbide A	C35 H36 N4 O5	cpd:C18021	HMDB0256449	NA	Organoheterocyclic compounds
Astragaloside IV	C41 H68 O14	cpd:C17799	NA	NA	Lipids and lipid-like molecules
Icariin	C33 H40 O15	cpd:C17555	NA	LMPK12112009	Phenylpropanoids and polyketides
Sweroside	C16 H22 O9	cpd:C17071	HMDB0258643	NA	Organic oxygen compounds
Methyl Palmitate	C17 H34 O2	cpd:C16995	HMDB0061859	NA	Lipids and lipid-like molecules
Ligustilide	C12 H14 O2	cpd:C16987	HMDB0034277	NA	Organoheterocyclic compounds
Hirsuteine	C22 H26 N2 O3	cpd:C16971	NA	NA	Alkaloids and derivatives
Arctiin	C27 H34 O11	cpd:C16915	HMDB0248568	NA	Lignans, neolignans and related compounds
Pentadecanoic Acid	C15 H30 O2	cpd:C16537	HMDB0000826	LMFA01010015	Lipids and lipid-like molecules
4-Hydroxybenzophenone	C13 H10 O2	cpd:C14230	HMDB0240708	NA	Benzenoids
Stearamide	C18 H37 N O	cpd:C13846	HMDB0034146	LMFA08010003	Organic acids and derivatives
Trifolin	C21 H20 O11	cpd:C12626	HMDB0030864	LMPK12111663	Phenylpropanoids and polyketides
Ecgonine methyl ester	C10 H17 N O3	cpd:C12448	HMDB0006406	NA	Alkaloids and derivatives
Mupirocin	C26 H44 O9	cpd:C11758	HMDB0014554	NA	Lipids and lipid-like molecules
Methyl jasmonate	C13 H20 O3	cpd:C11512	HMDB0036583	LMFA02020010	Lipids and lipid-like molecules
Carbendazim	C9 H9 N3 O2	cpd:C10897	HMDB0031769	NA	Organoheterocyclic compounds
Trachelogenin	C21 H24 O7	cpd:C10891	HMDB0303743	NA	Lignans, neolignans and related compounds
lupinine	C10 H19 N O	cpd:C10773	HMDB0304704	NA	Alkaloids and derivatives

Ammothamnine	C15 H24 N2 O2	cpd:C10749	NA	NA	Alkaloids and derivatives
Dictamnine	C12 H9 N O2	cpd:C10660	HMDB0251209	NA	Organoheterocyclic compounds
Tectoridin	C22 H22 O11	cpd:C10533	NA	LMPK12050372	Phenylpropanoids and polyketides
Curcumin	C21 H20 O6	cpd:C10443	HMDB0002269	NA	Phenylpropanoids and polyketides
4-p-Coumaroylquinic acid	C16 H18 O8	cpd:C10441	HMDB0301710	NA	Organic oxygen compounds
5-O-Caffeoylshikimic acid	C16 H16 O8	cpd:C10434	HMDB0033999	NA	Phenylpropanoids and polyketides
Astringin	C20 H22 O9	cpd:C10245	HMDB0303169	LMPK13090007	Phenylpropanoids and polyketides
Pinocembrin	C15 H12 O4	cpd:C09827	HMDB0030808	LMPK12140214	Phenylpropanoids and polyketides
Benzamide	C7 H7 N O	cpd:C09815	HMDB0004461	NA	Benzenoids
Verbenalin	C17 H24 O10	cpd:C09802	HMDB0301853	LMPR0102070016	Lipids and lipid-like molecules
Gentiopicrin	C16 H20 O9	cpd:C09782	NA	LMPR0102070009	Lipids and lipid-like molecules
Genipin	C11 H14 O5	cpd:C09780	HMDB0035830	NA	Lipids and lipid-like molecules
Aucubin	C15 H22 O9	cpd:C09771	HMDB0036562	LMPR0102070006	Lipids and lipid-like molecules
Salsolinol	C10 H13 N O2	cpd:C09642	HMDB0042012	NA	Organoheterocyclic compounds
Stevioside	C38 H60 O18	cpd:C09189	HMDB0034945	LMPR01040119	Lipids and lipid-like molecules
Geranylgeraniol	C20 H34 O	cpd:C09094	NA	LMPR0104010009	Lipids and lipid-like molecules
Aloesin	C19 H22 O9	cpd:C08994	NA	NA	Organic oxygen compounds
Cucurbitacin I	C30 H42 O7	cpd:C08800	HMDB0250582	LMST01010110	Lipids and lipid-like molecules
Isoguanosine	C10 H13 N5 O5	cpd:C08432	NA	NA	Nucleosides, nucleotides, and analogues
Myristoleic Acid	C14 H26 O2	cpd:C08322	HMDB0002000	LMFA01030051	Lipids and lipid-like molecules
5-Methoxy-N,N-dimethyltryptamine	C13 H18 N2 O	cpd:C08309	HMDB0002004	NA	Organoheterocyclic compounds

Hordatine B	C29 H40 N8 O5	cpd:C08308	HMDB0030459	NA	Phenylpropanoids and polyketides
Hordatine A	C28 H38 N8 O4	cpd:C08307	HMDB0030461	NA	Phenylpropanoids and polyketides
Octanedioic acid	C8 H14 O4	cpd:C08278	HMDB0000893	NA	Lipids and lipid-like molecules
Cefdinir	C14 H13 N5 O5 S2	cpd:C08110	HMDB0014675	NA	Organoheterocyclic compounds
Oxymorphone	C17 H19 N O4	cpd:C08019	HMDB0015323	NA	Benzenoids
Gossypol	C30 H30 O8	cpd:C07667	HMDB0040723	LMPR0103330002	Lipids and lipid-like molecules
Bilobalide	C15 H18 O8	cpd:C07605	HMDB0242203	NA	Lipids and lipid-like molecules
Amikacin	C22 H43 N5 O13	cpd:C06820	HMDB0014622	NA	Organoheterocyclic compounds
Nornicotine	C9 H12 N2	cpd:C06524	HMDB0001126	NA	Organoheterocyclic compounds
Eicosapentaenoic acid	C20 H30 O2	cpd:C06428	HMDB0001999	NA	Lipids and lipid-like molecules
Gamma-Linolenic Acid	C18 H30 O2	cpd:C06426	HMDB0003073	LMFA01030141	Lipids and lipid-like molecules
Terephthalic acid	C8 H6 O4	cpd:C06337	HMDB0002428	NA	Organic acids and derivatives
Salidroside	C14 H20 O7	cpd:C06046	HMDB0257463	NA	Organic oxygen compounds
Kaempferol	C15 H10 O6	cpd:C05903	HMDB0005801	LMPK12110003	Phenylpropanoids and polyketides
Coumarin	C9 H6 O2	cpd:C05851	HMDB0001218	NA	Phenylpropanoids and polyketides
Rutin	C27 H30 O16	cpd:C05625	HMDB0003249	LMPK12112098	Phenylpropanoids and polyketides
Homovanillic acid	C9 H10 O4	cpd:C05582	HMDB0000118	NA	Benzenoids
Nicotianamine	C12 H21 N3 O6	cpd:C05324	HMDB0255025	NA	Organic acids and derivatives
Estriol	C18 H24 O3	cpd:C05141	HMDB0000153	LMST02010003	Lipids and lipid-like molecules
Phenylacetylglutamine	C13 H16 N2 O4	cpd:C04148	HMDB0006344	NA	Organic acids and derivatives
Dodecanedioic acid	C12 H22 O4	cpd:C02678	HMDB0000623	LMFA01170009	Lipids and lipid-like molecules
5-Methylcytosine	C5 H7 N3 O	cpd:C02376	HMDB0002894	NA	Organoheterocyclic compounds
trans-Aconitic acid	C6 H6 O6	cpd:C02341	HMDB0000958	NA	Organic acids and derivatives
1-Methyladenine	C6 H7 N5	cpd:C02216	HMDB0011599	NA	Organoheterocyclic compounds
Glycyl-L-leucine	C8 H16 N2 O3	cpd:C02155	HMDB0028929	NA	Organic acids and derivatives

Phenylglyoxylic acid	C8 H6 O3	cpd:C02137	HMDB0001587	NA	Benzenoids
L-Pyroglutamic acid	C5 H7 N O3	cpd:C01879	HMDB0000267	NA	Organic acids and derivatives
Quercitrin	C21 H20 O11	cpd:C01750	HMDB0033751	LMPK12112171	Phenylpropanoids and polyketides
Methionine	C5 H11 N O2 S	cpd:C01733	HMDB0033951	NA	Organic acids and derivatives
Isovitexin	C21 H20 O10	cpd:C01714	HMDB0253708	LMPK12110338	Phenylpropanoids and polyketides
Vindoline	C25 H32 N2 O6	cpd:C01626	HMDB0259814	NA	Alkaloids and derivatives
2-Furoic acid	C5 H4 O3	cpd:C01546	HMDB0000617	NA	Organoheterocyclic compounds
Eleutheroside B	C17 H24 O9	cpd:C01533	NA	NA	Organic oxygen compounds
Geraniol	C10 H18 O	cpd:C01500	HMDB0005812	NA	Lipids and lipid-like molecules
4-Methylphenol	C7 H8 O	cpd:C01468	HMDB0001858	NA	Benzenoids
Ouabain	C29 H44 O12	cpd:C01443	HMDB0015224	LMST01120022	Lipids and lipid-like molecules
5-Hydroxytryptophan	C11 H12 N2 O3	cpd:C01017	HMDB0000472	NA	Organoheterocyclic compounds
Pantetheine	C11 H22 N2 O4 S	cpd:C00831	HMDB0003426	NA	Organic acids and derivatives
D-Glucarate	C6 H10 O8	cpd:C00818	HMDB0000663	NA	Organic oxygen compounds
Salicylic acid	C7 H6 O3	cpd:C00805	HMDB0001895	NA	Benzenoids
Phloretin	C15 H14 O5	cpd:C00774	HMDB0003306	LMPK12120525	Phenylpropanoids and polyketides
Coniferin	C16 H22 O8	cpd:C00761	HMDB0013682	NA	Organic oxygen compounds
Prostaglandin D2	C20 H32 O5	cpd:C00696	HMDB0001403	LMFA03010004	Lipids and lipid-like molecules
Estrone	C18 H22 O2	cpd:C00468	HMDB0000145	LMST02010004	Lipids and lipid-like molecules
Indole	C8 H7 N	cpd:C00463	HMDB0000738	NA	Organoheterocyclic compounds
trans-Cinnamic acid	C9 H8 O2	cpd:C00423	HMDB0000930	NA	Phenylpropanoids and polyketides
Tryptamine	C10 H12 N2	cpd:C00398	HMDB0000303	NA	Organoheterocyclic compounds
Gamma-Aminobutyric acid	C4 H9 N O2	cpd:C00334	HMDB0000112	NA	Organic acids and derivatives
L-Citrulline	C6 H13 N3 O3	cpd:C00327	HMDB0000904	NA	Organic acids and derivatives
Nicotinic acid	C6 H5 N O2	cpd:C00253	HMDB0001488	NA	Organoheterocyclic compounds
Guanine	C5 H5 N5 O	cpd:C00242	HMDB0000132	NA	Organoheterocyclic compounds

Adenosine	C10 H13 N5 O4	cpd:C00212	HMDB0000050	NA	Nucleosides, nucleotides, and analogues
L-Threonine	C4 H9 N O3	cpd:C00188	HMDB0000167	NA	Organic acids and derivatives
Citric acid	C6 H8 O7	cpd:C00158	HMDB0000094	NA	Organic acids and derivatives
Adenine	C5 H5 N5	cpd:C00147	HMDB0000034	NA	Organoheterocyclic compounds
3-Methyl-2-Oxobutanoic Acid	C5 H8 O3	cpd:C00141	HMDB0030027	NA	Organic acids and derivatives
L-Histidine	C6 H9 N3 O2	cpd:C00135	HMDB0000177	NA	Organic acids and derivatives
S-Adenosylhomocysteine	C14 H20 N6 O5 S	cpd:C00021	HMDB0000939	NA	Nucleosides, nucleotides, and analogues
S-Adenosylmethionine	C15 H22 N6 O5 S	cpd:C00019	HMDB0001185	NA	Nucleosides, nucleotides, and analogues
Estrone sulfate	C18 H22 O5 S	NA	HMDB0001425	LMST02010043	Lipids and lipid-like molecules
Hesperetin 5-O-glucoside	C22 H24 O11	NA	HMDB0037535	LMPK12140377	Phenylpropanoids and polyketides
Trilobatin	C21 H24 O10	NA	HMDB0037505	LMPK12120518	Phenylpropanoids and polyketides
Typhaneoside	C34 H42 O20	NA	HMDB0039541	LMPK12112350	Phenylpropanoids and polyketides
7-Methoxyflavone	C16 H12 O3	NA	NA	LMPK12110021	Phenylpropanoids and polyketides
Isomucronulatol	C17 H18 O5	NA	HMDB0033189	LMPK12080033	Phenylpropanoids and polyketides
Epigallocatechin	C15 H14 O7	NA	HMDB0038365	LMPK12020004	Phenylpropanoids and polyketides
LPI 18:1	C27 H51 O12 P	NA	NA	LMGP06050005	Lipids and lipid-like molecules
LPG 18:4	C24 H41 O9 P	NA	NA	LMGP04050021	Lipids and lipid-like molecules
LPG 15:1	C21 H41 O9 P	NA	NA	LMGP04050018	Lipids and lipid-like molecules
LPG 16:0	C22 H45 O9 P	NA	NA	LMGP04050008	Lipids and lipid-like molecules
LPE 18:3	C23 H42 N O7 P	NA	NA	LMGP02050043	Lipids and lipid-like molecules
LPC 18:3	C26 H48 N O7 P	NA	NA	LMGP01050128	Lipids and lipid-like molecules
gamma-caprolactone	C6 H10 O2	NA	HMDB0003843	LMFA07040010	Organoheterocyclic compounds
Avocadyne 1-acetate	C19 H34 O4	NA	HMDB0031048	LMFA05000644	Lipids and lipid-like molecules
Prostaglandin K2	C20 H30 O5	NA	NA	LMFA03010023	Lipids and lipid-like molecules
Tetranor-12R-HETE	C16 H26 O3	NA	NA	LMFA01050143	Lipids and lipid-like molecules
Heptadecanoic Acid	C17 H34 O2	NA	HMDB0002259	LMFA01010017	Lipids and lipid-like molecules

P-Aminohippuric Acid	C9 H10 N2 O3	NA	HMDB0001867	NA	Benzenoids
2-Hydroxyisocaproic Acid	C6 H12 O3	NA	HMDB0000665	NA	Lipids and lipid-like molecules
3-Indolepropionic acid	C11 H11 N O2	NA	HMDB0002302	NA	Organoheterocyclic compounds
(±)-Abscisic acid	C15 H20 O4	NA	NA	NA	Lipids and lipid-like molecules
N-{4-[(2R,3R)-3-(Hydroxymethyl)-5-oxo-2-morpholinyl]phenyl}acetamide	C13 H16 N2 O4	NA	NA	NA	Organoheterocyclic compounds
Sattabacin	C13 H18 O2	NA	NA	NA	Benzenoids
Yuheinoside	C16 H24 O9	NA	NA	NA	Lipids and lipid-like molecules
5-[(Benzoyloxy)methyl]-4,5,6-trihydroxy-2-cyclohexen-1-yl benzoate	C21 H20 O7	NA	NA	NA	Benzenoids
Estriol 17-sulfate	C18 H24 O6 S	NA	NA	NA	Lipids and lipid-like molecules
Zeatin-7-N-glucoside	C16 H23 N5 O6	NA	NA	NA	Organic oxygen compounds
Heteroclitin D	C27 H30 O8	NA	NA	NA	Phenylpropanoids and polyketides
δ-Ribono-1,4-lactone	C5 H8 O5	NA	NA	NA	Organic oxygen compounds
N-Sinapoylputrescine	C15 H22 N2 O4	NA	NA	NA	Benzenoids
Artesunate	C19 H28 O8	NA	HMDB0240267	NA	Lipids and lipid-like molecules
6-Methylquinoline	C10 H9 N	NA	HMDB0033115	NA	Organoheterocyclic compounds
Sesamoside	C17 H24 O12	NA	NA	NA	Organic oxygen compounds
N-lactoyl-phenylalanine	C12 H15 N O4	NA	HMDB0062175	NA	Organic acids and derivatives
8-Epiloganic acid	C16 H24 O10	NA	HMDB0247433	NA	Lipids and lipid-like molecules
3-(3,4-dihydroxyphenyl)propanoic acid	C9 H10 O4	NA	HMDB0000423	NA	Phenylpropanoids and polyketides
Methyl dihydrojasmonate	C13 H22 O3	NA	HMDB0031740	NA	Lipids and lipid-like molecules

Ajugol	C15 H24 O9	NA	HMDB0248074	NA	Lipids and lipid-like molecules
4-((5-(4-Nitrophenyl)oxazol-2-yl)amino)benzonitrile	C16 H10 N4 O3	NA	NA	NA	Organoheterocyclic compounds
Deoxycorticosterone 21-glucoside	C27 H40 O8	NA	NA	NA	Lipids and lipid-like molecules
L-Glutathione oxidized	C20 H32 N6 O12 S2	NA	NA	NA	Organic acids and derivatives
2-[(3S)-1-Isopropyl-3-pyrrolidinyl]-5-methyl-1H-benzimidazole	C15 H21 N3	NA	NA	NA	Organoheterocyclic compounds
Pinoresinol Diglucoside	C32 H42 O16	NA	NA	NA	Lignans, neolignans and related compounds
Prolylleucine	C11 H20 N2 O3	NA	HMDB0253028	NA	Organic acids and derivatives
N'-[4-(trifluoromethyl)benzoyl]-6-quinoxalinecarbohydrazide	C17 H11 F3 N4 O2	NA	NA	NA	Organic acids and derivatives
Nuzhenide	C31 H42 O17	NA	HMDB0302806	NA	Lipids and lipid-like molecules
O-Acetylserine	C5 H9 N O4	NA	HMDB0003011	NA	Organic acids and derivatives
Vardenafil N-oxide	C23 H32 N6 O5 S	NA	NA	NA	Benzenoids
Dinophysistoxin-1	C45 H70 O13	NA	HMDB0030442	NA	Organic oxygen compounds
Tenuifoliside B	C30 H36 O17	NA	NA	NA	Phenylpropanoids and polyketides
Uridine 5'-diphosphogalactose	C15 H24 N2 O17 P2	NA	HMDB0012305	NA	Nucleosides, nucleotides, and analogues
Madecassic acid	C30 H48 O6	NA	HMDB0036670	NA	Lipids and lipid-like molecules
α-Linolenoyl ethanolamide	C20 H35 N O2	NA	HMDB0013624	NA	Organic nitrogen compounds
DL-Tryptophan	C11 H12 N2 O2	NA	HMDB0030396	NA	Organoheterocyclic compounds
20-Hydroxy-(5Z,8Z,11Z,14Z)-eicosatetraenoic acid	C20 H32 O3	NA	HMDB0005998	NA	Lipids and lipid-like molecules

Dihomo-γ-linolenic acid methyl ester	C21 H36 O2	NA	NA	NA	Lipids and lipid-like molecules
Nicotinate ribonucleoside	C11 H13 N O6	NA	HMDB0006809	NA	Organic oxygen compounds
Acetylharpagide	C17 H26 O11	NA	NA	NA	Lipids and lipid-like molecules
Homoveratrumic acid	C10 H12 O4	NA	HMDB0000434	NA	Benzenoids
8(9)-EET Ethanolamide	C22 H37 N O3	NA	NA	NA	Organic nitrogen compounds
Ala-Ile	C9 H18 N2 O3	NA	HMDB0028690	NA	Organic acids and derivatives
Sclareolide	C16 H26 O2	NA	HMDB0035293	NA	Organoheterocyclic compounds
O-Feruloyl 4-hydroxylcoumarin	C19 H14 O6	NA	NA	NA	Organic oxygen compounds
D-(-)-Glutamine	C5 H10 N2 O3	NA	NA	NA	Organic acids and derivatives
Methyl linolenate	C19 H32 O2	NA	HMDB0254600	NA	Lipids and lipid-like molecules
tert-Butyl N-[1-(aminocarbonyl)-3-methylbutyl]carbamate	C11 H22 N2 O3	NA	NA	NA	Organic acids and derivatives
2-Hydroxy-2-methylbutanoic acid	C5 H10 O3	NA	HMDB0001987	NA	Lipids and lipid-like molecules
4-Toluenesulfonic acid	C7 H8 O3 S	NA	HMDB0059933	NA	Benzenoids
N-Acetyl-DL-tryptophan	C13 H14 N2 O3	NA	NA	NA	Organic acids and derivatives
Azilsartan	C25 H20 N4 O5	NA	HMDB0248802	NA	Benzenoids
Coenzyme Q2	C19 H26 O4	NA	HMDB0006709	NA	Lipids and lipid-like molecules
Indole-3-pyruvic acid	C11 H9 N O3	NA	HMDB0060484	NA	Organoheterocyclic compounds
2-Hydroxyhippuric acid	C9 H9 N O4	NA	HMDB0000840	NA	Benzenoids
Sophocarpine	C15 H22 N2 O	NA	HMDB0258379	NA	Alkaloids and derivatives
Anise oil	C14 H18 O3	NA	NA	NA	Benzenoids

N-Acetyl-5-aminosalicylic acid	C9 H9 N O4	NA	HMDB0060602	NA	Benzenoids
Choline	C5 H13 N O	NA	HMDB0000097	NA	Organic nitrogen compounds
Diosbulbin B	C19 H20 O6	NA	HMDB0036777	NA	Organoheterocyclic compounds
Neohesperidin dihydrochalcone	C28 H36 O15	NA	HMDB0030542	NA	Phenylpropanoids and polyketides
Hastatoside	C17 H24 O11	NA	HMDB0301945	NA	Lipids and lipid-like molecules
all-cis-4,7,10,13,16-Docosapentaenoic acid	C22 H34 O2	NA	NA	NA	Lipids and lipid-like molecules
9-Oxo-10(E),12(E)-octadecadienoic acid	C18 H30 O3	NA	NA	NA	Lipids and lipid-like molecules
N'-Formylkynurenine	C11 H12 N2 O4	NA	HMDB0001200	NA	Organic oxygen compounds
methyl oxo pentanoate	C6 H10 O3	NA	NA	NA	Organic acids and derivatives
Phosphocholine	C5 H14 N O4 P	NA	HMDB0001565	NA	Organic nitrogen compounds
Curcumol	C15 H24 O2	NA	HMDB0038122	NA	Lipids and lipid-like molecules
Cotinine	C10 H12 N2 O	NA	HMDB0001046	NA	Organoheterocyclic compounds
1-Linoleoyl-Rac-Glycerol	C21 H38 O4	NA	NA	NA	Lipids and lipid-like molecules
5-Hydroxyindole-3-acetic acid	C10 H9 N O3	NA	HMDB0000763	NA	Organoheterocyclic compounds
N-Isovaleroylglycine	C7 H13 N O3	NA	HMDB0000678	NA	Organic acids and derivatives
MAG (18:3)	C21 H36 O4	NA	NA	NA	Lipids and lipid-like molecules
Diflucortolone pivalate	C27 H36 F2 O5	NA	NA	NA	Lipids and lipid-like molecules
Myrtillin chloride	C21 H21 Cl O12	NA	NA	NA	Phenylpropanoids and polyketides
(1R,2S,3R,4R)-3-(Isobutylamino)-4-(3-pyridinyl)-1,2-cyclopentanediol	C14 H22 N2 O2	NA	NA	NA	Organic nitrogen compounds

Kirenol	C20 H34 O4	NA	HMDB0253803	NA	Lipids and lipid-like molecules
Diethyl succinate	C8 H14 O4	NA	HMDB0033838	NA	Lipids and lipid-like molecules
Ziyuglycoside II	C35 H56 O8	NA	NA	NA	Lipids and lipid-like molecules
Tyrosylalanine	C12 H16 N2 O4	NA	HMDB0029098	NA	Organic acids and derivatives
Denin	C33 H56 O14	NA	NA	NA	Lipids and lipid-like molecules
2-(14,15-Epoxyeicosatrienoyl) glycerol	C23 H38 O5	NA	HMDB0013651	NA	Lipids and lipid-like molecules
13,14-dihydro Prostaglandin F1α	C20 H38 O5	NA	NA	NA	Lipids and lipid-like molecules
N-Lauroylsarcosine	C15 H29 N O3	NA	HMDB0255165	NA	Organic acids and derivatives
Quercetin 3-alpha-L-arabinofuranoside (Avicularin)	C20 H18 O11	NA	NA	NA	Phenylpropanoids and polyketides
Tubuloside A	C37 H48 O21	NA	NA	NA	Organic oxygen compounds
Norisoboldine	C18 H19 N O4	NA	HMDB0033357	NA	Alkaloids and derivatives
N-{[(2R,4S,5R)-5-Ethyl-1-azabicyclo[2.2.2]oct-2-yl]methyl}-2-furamide	C15 H22 N2 O2	NA	NA	NA	Organoheterocyclic compounds
TriacetonaMine	C9 H17 N O	NA	HMDB0031179	NA	Organoheterocyclic compounds
Toddalolactone	C16 H20 O6	NA	NA	NA	Phenylpropanoids and polyketides
(R,S)-Anatabine	C10 H12 N2	NA	NA	NA	Organic nitrogen compounds
Guanosine monophosphate (GMP)	C10 H14 N5 O8 P	NA	NA	NA	Nucleosides, nucleotides, and analogues
(+)-Isolariciresinol	C20 H24 O6	NA	HMDB0301739	NA	Lignans, neolignans and related compounds
Dl-Indole-3-lactic acid	C11 H11 N O3	NA	NA	NA	Organoheterocyclic compounds
Alanyltyrosine	C12 H16 N2 O4	NA	HMDB0028699	NA	Organic acids and derivatives
(+/-)9-HpODE	C18 H32 O4	NA	NA	NA	Lipids and lipid-like molecules

L-5-Hydroxytryptophan	C11 H12 N2 O3	NA	HMDB0000472	NA	Organoheterocyclic compounds
Tetranor-12(S)-HETE	C16 H26 O3	NA	NA	NA	Lipids and lipid-like molecules
1,1-Dimethyl-2-oxopropyl N-[2-(2-pyridyl)ethyl]carbamate	C13 H18 N2 O3	NA	NA	NA	Organoheterocyclic compounds
Methyl linoleate	C19 H34 O2	NA	HMDB0034381	NA	Lipids and lipid-like molecules
Neodiosmin	C28 H32 O15	NA	HMDB0039857	NA	Phenylpropanoids and polyketides
Gly-Phe	C11 H14 N2 O3	NA	HMDB0028848	NA	Organic acids and derivatives
Indole-3-acrylic acid	C11 H9 N O2	NA	HMDB0000734	NA	Organoheterocyclic compounds
N-(p-Coumaroyl) serotonin	C19 H18 N2 O3	NA	HMDB0255027	NA	Organoheterocyclic compounds
Ziyuglycoside I	C41 H66 O13	NA	NA	NA	Lipids and lipid-like molecules
α-Hydroxyhippuric acid	C9 H9 N O4	NA	NA	NA	Benzenoids
16,16-Dimethyl prostaglandin A1	C22 H36 O4	NA	NA	NA	Lipids and lipid-like molecules
Maltotriitol	C18 H34 O16	NA	NA	NA	Organic oxygen compounds
Mogroside III	C48 H82 O19	NA	HMDB0254831	NA	Lipids and lipid-like molecules
N-Desmethylclobazam	C15 H11 Cl N2 O2	NA	HMDB0060970	NA	Organoheterocyclic compounds
2-Ethylbutyric Acid	C6 H12 O2	NA	HMDB0031221	NA	Lipids and lipid-like molecules
12-oxo Phytodienoic Acid	C18 H28 O3	NA	NA	NA	Lipids and lipid-like molecules
Quercetin-3β-D-glucoside	C21 H20 O12	NA	NA	NA	Phenylpropanoids and polyketides
Timosaponin A1	C33 H54 O8	NA	NA	NA	Lipids and lipid-like molecules
N-Acetyl-L-Tyrosine	C11 H13 N O4	NA	HMDB0000866	NA	Organic acids and derivatives
Androsin	C15 H20 O8	NA	NA	NA	Lipids and lipid-like molecules
Thromoboxane B1	C20 H36 O6	NA	NA	NA	Lipids and lipid-like molecules
Polyphyllin V	C39 H62 O12	NA	NA	NA	Lipids and lipid-like molecules
beta,beta-Dimethylacrylalkannin	C21 H22 O6	NA	NA	NA	Benzenoids

Verrucarol	C15 H22 O4	NA	NA	NA	Lipids and lipid-like molecules
Adenosine 3'5'-cyclic monophosphate	C10 H12 N5 O6 P	NA	NA	NA	Nucleosides, nucleotides, and analogues
7-O-Ethylmorroniside	C19 H30 O11	NA	NA	NA	Organic oxygen compounds
PDMP	C23 H38 N2 O3	NA	NA	NA	Organic nitrogen compounds
Methylhippuric acid	C10 H11 N O3	NA	HMDB0255085	NA	Organic acids and derivatives
N-Feruloyl putrescine	C14 H20 N2 O3	NA	NA	NA	Phenylpropanoids and polyketides
Angelol B	C20 H24 O7	NA	HMDB0248421	NA	Phenylpropanoids and polyketides
